# Chronic lead exposure and burden of cardiovascular disease during 1990–2019: a systematic analysis of the global burden of disease study

**DOI:** 10.3389/fcvm.2024.1367681

**Published:** 2024-04-09

**Authors:** Peizhu Dang, Manyun Tang, Heze Fan, Junjun Hao

**Affiliations:** ^1^ Department of Cardiovascular Medicine, The First Affiliated Hospital of Xi'an Jiaotong University, Xi’an, China; ^2^ Department of Hepatobiliary Surgery, The First Affiliated Hospital of Xi'an Jiaotong University, Xi’an, China; ^3^ Department of Cardiovascular Surgery, The First Affiliated Hospital of Xi'an Jiaotong University, Xi’an, China

**Keywords:** chronic lead exposure, cardiovascular disease, mortality, disability-adjusted life-years, age-standardized rates

## Abstract

**Background:**

Cardiovascular diseases (CVD) are the leading causes of death and disability worldwide. Lead exposure is an important risk factor for CVD. In our study, we aimed to estimate spatial and temporal trends in the burden of cardiovascular disease associated with chronic lead exposure.

**Methods:**

The data collected for our study were obtained from Global Burden of Disease (GBD) study 2019 and analyzed by age, sex, cause, and location. To assess the temporal trends in burden of CVD attributable to chronic lead exposure over 30 years, we used Joinpoint regression analysis.

**Results:**

In 2019, the number of lead exposure-attributable CVD deaths and disability-adjusted life-years (DALYs) were 0.85 and 17.73 million, 1.7 and 1.4 times more than those observed in 1990, respectively. However, the corresponding age-standardized rates (ASR) of death and DALY gradually decreased from 1990 to 2019, especially from 2013 to 2019. Over the last 30 years, among 21 GBD regions and 204 countries and territories, the High-income Asia Pacific and the Republic of Korea experienced the largest reductions in age-standardized DALY and death rates, while Central Asia and Afghanistan experienced the largest increases. Males and the elderly population suffered higher death rates and DALY burdens than females and the young population. Furthermore, we observed that higher socio-demographic index (SDI) regions demonstrated lower ASR of death and DALY rates. In 2019, the low and low-middle SDI regions, especially South Asia, exhibited the highest burden of CVD attributable to lead exposure.

**Conclusion:**

Our study provides a thorough understanding of the burden of CVD attributable to chronic lead exposure. The findings confirm the significance of implementing lead mitigation strategies and increasing investment in CVD prevention and treatment. These measures are crucial in reducing the burden of CVD and promoting public health on a global scale.

## 1 Introduction

Cardiovascular diseases (CVD) are the leading cause of mortality and a significant contributor to disability worldwide. According to the 2019 update of the global burden of disease (GBD) study, the total global mortality attributed to CVD continued to increase from 12.1 million in 1990 to 18.6 million in 2019 (1). Although often overlooked, environmental exposure is a crucial risk factor for CVD (2). Lead, primarily from industrial sources but still present in paints and fuels in some countries (3), contributes to nearly one million deaths and 21.7 million disability-adjusted life-years (DALY) globally (4). Unlike blood lead with a 30-day half-life, bone lead, lasting years to decades, more accurately indicates long-term exposure and accumulation.

Lead exposure is recognized as a risk factor for the incidence and mortality of CVD (4–6). Studies have reported that even at low levels, lead exposure can increase the incidence of hypertension, stroke, and other CVD. Emerging evidence has demonstrated an association between bone lead and resistant hypertension (7). A meta-analysis revealed that a 10 µg/g increase in bone lead was associated with a 0.26 mmHg increase in systolic blood pressure (SBP) (95% CI: 0.02–0.50) (8). In addition, Nitin B. Jain et al. reported that the group with increased bone lead levels were at higher risk for future ischemic heart disease (IHD), for the reason that lead exposure can damage the inner lining of blood vessels, leading to inflammation and the formation of plaque, which can cause the narrowing of blood vessels (9). Yang et al., in their 2017 prospective study on a population cohort, revealed a notable decrease in the left ventricle's systolic function due to environmental lead exposure (10).

Understanding the burden of CVD caused by chronic lead exposure is key for implementing prevention strategies, a comprehensive study is needed to provide a clear picture of the situation. Fortunately, the GBD Study 2019 has identified lead exposure as a risk factor for CVD. Therefore, we estimated spatial and temporal trends in the burden of lead-related CVD and identified heavily affected regions based on the latest data from GBD 2019 and improved methods. This information is vital for government and health system decision-making regarding the implementation of effective prevention and mitigation strategies.

## 2 Methods

### 2.1 Data source

The data on the global burden of CVD attributable to lead exposure was obtained from GBD 2019, and the detailed methods of data collection, processing, and modeling methods were fully explained in previous studies (11). In brief, GBD 2019 is a comprehensive and scientific assessment of the global disease, injury, and risk factors affecting individuals of different ages and genders. A total of 204 countries or territories were included, and according to epidemiological homogeneity and geographical proximity, they were grouped into 21 regions. And they were divided into five groups according to the socio-demographic index (SDI), a composite indicator based on per capita income, average educational attainment and total fertility rate. We used the GBD 2019 database to extract information on the number of deaths, DALY, and corresponding age-standardized rates (ASR) attributable to lead exposure from 1990 to 2019. The data was analyzed by age, gender, location and year to comprehensively understand the burden of CVD caused by lead exposure.

### 2.2 Definitions of CVD and lead exposure

According to the International Classification of Diseases (ICD) system, CVD is defined as encompassing 11 major cause categories: aortic aneurysm, atrial fibrillation, and flutter, cardiomyopathy and myocarditis, endocarditis, hypertensive heart disease (HHD), IHD, non-rheumatic valvular heart disease, lower extremity peripheral arterial disease, rheumatic heart disease, stroke and other cardiovascular and circulatory diseases (12, 13). IHD encompassed a range of conditions including acute myocardial infarction, chronic stable angina, chronic IHD, and heart failure resulting from IHD. Stroke was categorized based on the World Health Organization's criteria, and its estimates were further divided into three subcategories: ischemic stroke (IS), intracerebral hemorrhage, and subarachnoid hemorrhage. HHD was characterized as heart failure with symptoms directly and chronically induced by hypertension. Chronic lead exposure was measured as micrograms of lead per gram of bone (µg/g). The U.S. Centers for Disease Control and Prevention (CDC) set the blood lead reference value for children at 3.5 μg/dl and for adults at 5 μg/dl, updated from 5 μg/dl to 3.5 μg/dl in 2021 (4, 14).

### 2.3 Estimation of exposure to lead and Its attributable disease burden

Since 2010, the GBD studies have used the World Cancer Research Fund criteria for convincing or probable evidence of risk outcome pairs. To estimate bone lead exposure, a cumulative blood lead index was calculated for cohorts using estimated blood lead exposure over their lifetime (1). The cumulative blood lead index was then used to estimate bone lead by applying a scalar defined in literature. The blood lead index was calculated as follows: first, data on blood lead exposure were primarily extracted from literature reports and a few surveys in addition to a few blood lead surveys. Second, using the exposure distributions of blood lead over time and space, lifetime blood lead could be expressed as a curve over each year of life. To estimate bone lead in a given year, the cumulative blood lead index was calculated as the area under the curve and then used with the scalar to estimate bone lead. In GBD 2019, global exposure was modelled using the spatiotemporal Gaussian process regression methodology (ST-GPR). The GBD 2019 Risk Factors Collaborators calculated the population attributable fraction (PAF) for bone lead exposure and its paired outcomes using exposure estimates and relative risks, using the standard GBD PAF equation (1). As bone lead exposure is associated with an increase in SBP, all health burden attributable to bone lead exposure is mediated through SBP. The relative risks for bone lead exposure are the same as those for SBP and its outcomes. Therefore, bone lead level is paired with SBP, which in turn is related to all cardiovascular outcomes associated with SBP. The detail methods can be found in Supplementary Methods Description. Death and DALYs due to the specific causes were estimated using the standard method, as described in GBD 2019 report (1, 11) and the official website (http://www.healthdata.org/gbd/).

### 2.4 Statistical analysis

The burden of disease attributable to chronic lead exposure on CVD was analyzed by age, sex, year, and location. To remove the effects caused by differences in population structures, the ASR of death and DALY were used to compare different populations or the same population over different periods. The number of deaths, DALY, and ASR attributable to lead exposure were reported with 95% uncertainty intervals (UIs). Statistical analyses were conducted using R (version 4.1).

To evaluate the temporal trends in age-standardized death and DALY rates, the Joinpoint regression model was used. Briefly, this model separates a straight regression line into several statistically significant trend segments, with each segment described using a linear model. The method aims to determine the optimal number of breakthrough points to assess significant changes in trends over time (15). Joinpoint regression analysis was conducted using the Joinpoint software (version 4.7.0) from the Surveillance Research Program of the US National Cancer Institute. The annual percentage changes (APC) and their 95% confidence intervals were also calculated. The *P *< 0.05 was considered as statistical significance.

## 3 Results

### 3.1 Global burden of cardiovascular diseases attributable to lead exposure in 2019

Globally in 2019, lead exposure led to 0.85 million (95% UI: 0.52–1.21) deaths and 17.73 million (95%UI: 10.49–25.66) DALYs attributed to CVD, which were 1.7 times and 1.4 times higher than those recorded in 1990, respectively. The ASR of death was 10.80 (95% UI: 6.56–15.50) per 100,000 population, and the ASR of DALY was 216.80 (95% UI: 128.35–314.82) per 100,000 population. In 2019, IHD accounted for the largest proportion of CVD related to lead exposure, contributing to 48.7% and 47.2% of deaths and DALYs, respectively. Stroke was the second most significant cause, contributing to 36.0% of deaths and 38.0% of DALYs attributable to lead exposure, followed by HHD, which accounted for 11.5% and 10.0% of deaths and DALYs, respectively. Other diseases, including atrial fibrillation and flutter, rheumatic heart disease, aortic aneurysm, were of less importance.

**Figure 1:**
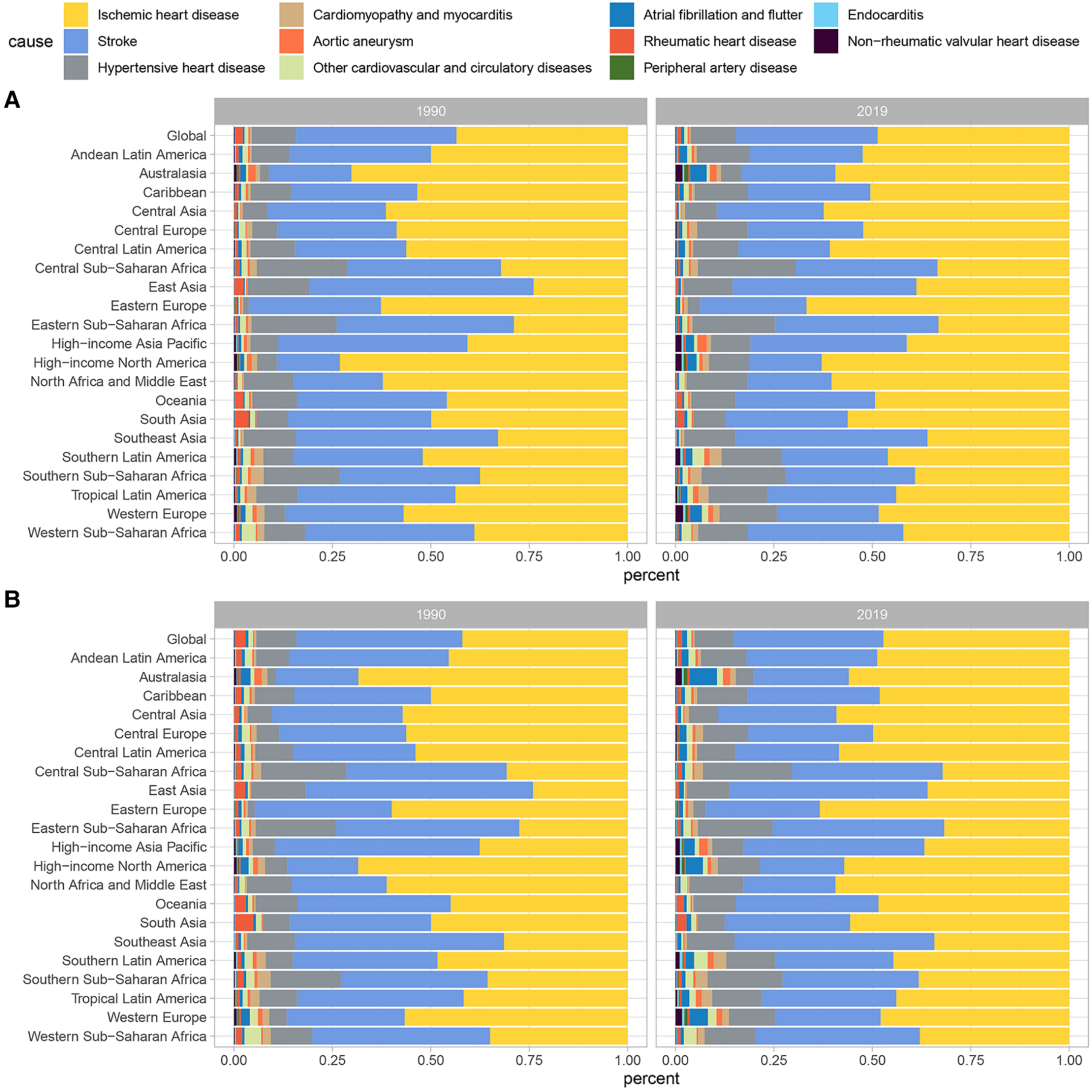
Contributions of specific CVD attributable to lead exposure to (**A**) death cases and (**B**) DALYs numbers in 1999 and 2019. CVD, cardiovascular diseases; DALY, disability-adjusted life year.

**Figure 2:**
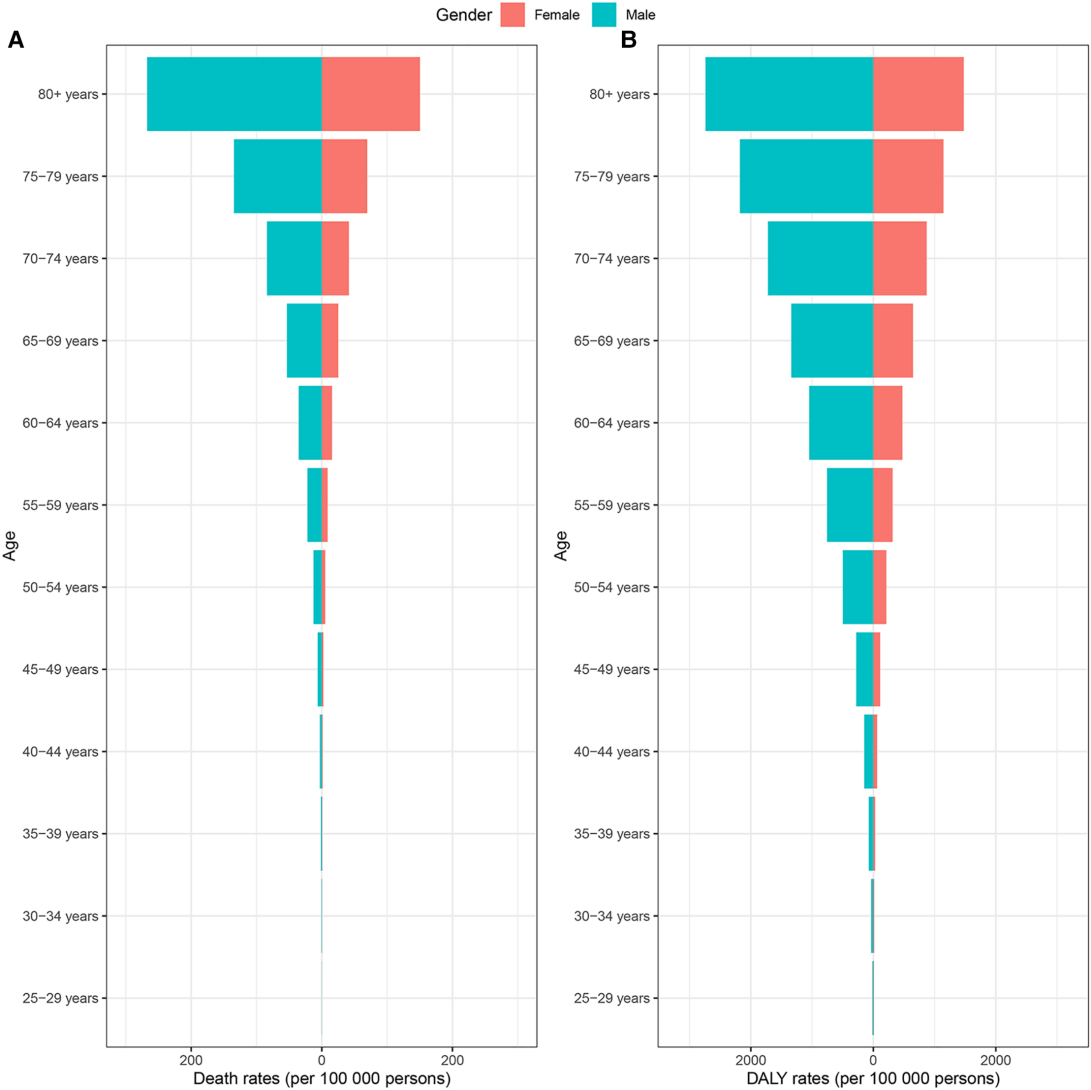
CVD burden attributable to lead exposure among different genders and ages in 2019. (**A**) Age-standardized death rates. (**B**) Age-standardized DALY rates. CVD, cardiovascular diseases; DALY, disability-adjusted life year.

At the global level, there were changes in the proportion of deaths and DALYs associated with specific CVD caused by lead exposure from 1990 to 2019, with the proportion of stroke decreasing and the proportion of IHD increasing (Table 1; Figure 1). The burden of CVD attributable to lead exposure showed significant heterogeneity between genders and different age groups. Males have a higher burden of CVD due to lead exposure compared to females (Figure 2). Moreover, age-specific rates of lead-related CVD DALY and death increased with increasing age in both genders.

**Table 1:** Global burden of cardiovascular diseases attributable to lead exposure in 1990 and 2019.

Outcomes	Diseases	Year	Number	95%UI		ASR (per 100,000 population)	95%UI	
				Upper	Lower		Upper	Lower
Deaths	All CVD	1990	508,453.26	745,947.43	299,712.32	13.89	20.55	7.99
		2019	848,777.75	1,212,097.67	517,041.94	10.80	15.50	6.56
	IHD	1990	220,991.64	334,743.65	124,894.91	6.12	9.46	3.36
		2019	413,040.44	615,745.19	242,839.05	5.26	7.88	3.08
	Stroke	1990	207,412.48	304,798.68	124,084.00	5.53	8.22	3.28
		2019	305,270.81	435,667.46	182,799.84	3.85	5.50	2.30
	HHD	1990	56,998.32	125,063.88	22,327.62	1.61	3.54	0.57
		2019	97,486.59	225,238.15	30,505.83	1.27	2.96	0.39
	RHD	1990	9,388.91	16,104.37	5,103.51	0.23	0.41	0.12
		2019	7,688.62	13,500.23	4,085.92	0.10	0.17	0.05
	Others CVD	1990	4,904.23	7,843.59	2,700.39	0.13	0.21	0.07
		2019	7,337.10	10,805.78	4,303.58	0.09	0.14	0.05
	CM	1990	2,557.15	4,515.40	1,052.78	0.08	0.14	0.03
		2019	3,476.84	6,353.81	1,449.28	0.04	0.08	0.02
	AF	1990	2,354.28	3,774.92	1,206.04	0.08	0.13	0.04
		2019	7,101.11	11,086.18	3,996.73	0.10	0.15	0.05
	Aortic aneurysm	1990	1,993.65	3,390.84	902.17	0.05	0.09	0.02
		2019	3,471.57	5,608.21	1,740.62	0.04	0.07	0.02
	Non-rheumatic VAD	1990	937.86	1,587.35	408.01	0.03	0.05	0.01
		2019	1,924.78	3,400.64	825.69	0.03	0.05	0.01
	Endocarditis	1990	613.32	1,016.10	312.67	0.02	0.03	0.01
		2019	1,194.47	2,091.68	554.47	0.02	0.03	0.01
	PAD	1990	301.41	679.08	102.56	0.01	0.02	0.00
		2019	785.42	1,682.96	295.30	0.01	0.02	0.00
DALY	All CVD	1990	12,884,534.84	18,646,607.86	7,733,967.95	318.54	461.21	190.85
		2019	17,734,897.91	25,660,400.92	10,487,787.87	216.80	314.82	128.35
	IHD	1990	5,408,631.30	7,950,628.87	3,178,311.05	134.35	199.09	78.06
		2019	8,368,695.85	12,449,232.13	4,896,397.71	102.26	152.87	59.74
	Stroke	1990	5,436,721.90	7,951,794.82	3,253,442.87	133.36	195.09	80.09
		2019	6,738,783.11	9,815,597.48	3,912,198.42	81.97	119.11	47.86
	HHD	1990	1,308,406.32	2,676,257.86	590,406.96	33.36	69.08	14.33
		2019	1,769,515.14	3,845,468.59	663,725.84	22.04	47.48	8.09
	RHD	1990	320,197.28	543,632.36	168,868.71	7.18	12.16	3.87
		2019	213,507.92	372,744.25	106,842.49	2.58	4.55	1.28
	Others CVD	1990	149,639.64	243,118.38	80,446.78	3.56	5.75	1.94
		2019	192,445.73	303,734.87	104,239.21	2.33	3.69	1.26
	CM	1990	62,114.40	108,093.94	25,650.36	1.57	2.70	0.65
		2019	74,481.93	136,563.45	29,416.20	0.92	1.68	0.36
	AF	1990	102,193.00	164,819.74	53,136.84	2.75	4.45	1.44
		2019	227,474.59	363,018.69	1,24,509.53	2.85	4.57	1.56
	Aortic aneurysm	1990	46,705.46	81,036.37	20,771.65	1.15	2.00	0.51
		2019	70,112.63	115,128.13	34,835.21	0.85	1.40	0.42
	Non-rheumatic VAD	1990	20,458.39	34,667.11	8,869.58	0.53	0.88	0.23
		2019	31,947.78	54,281.00	14,721.51	0.40	0.68	0.18
	Endocarditis	1990	19,634.09	33,380.20	9,570.01	0.45	0.74	0.22
		2019	27,921.46	50,456.24	12,559.69	0.34	0.62	0.15
	PAD	1990	9,833.05	19,082.78	3,917.30	0.27	0.54	0.11
		2019	20,011.80	37,726.73	8,648.14	0.25	0.48	0.11

ASR, age-standardized rates; DALY, disability-adjusted life-years; AF, atrial fibrillation, and flutter; CVD, cardiovascular diseases; CM, cardiomyopathy and myocarditis; HHD, hypertensive heart disease; IHD, ischemic heart disease (IHD); VAD, valvular heart disease; PAD, peripheral arterial disease; RHD, rheumatic heart disease.

### 3.2 Temporal trends of cardiovascular diseases burdens attributable to lead exposure

**Figure 3:**
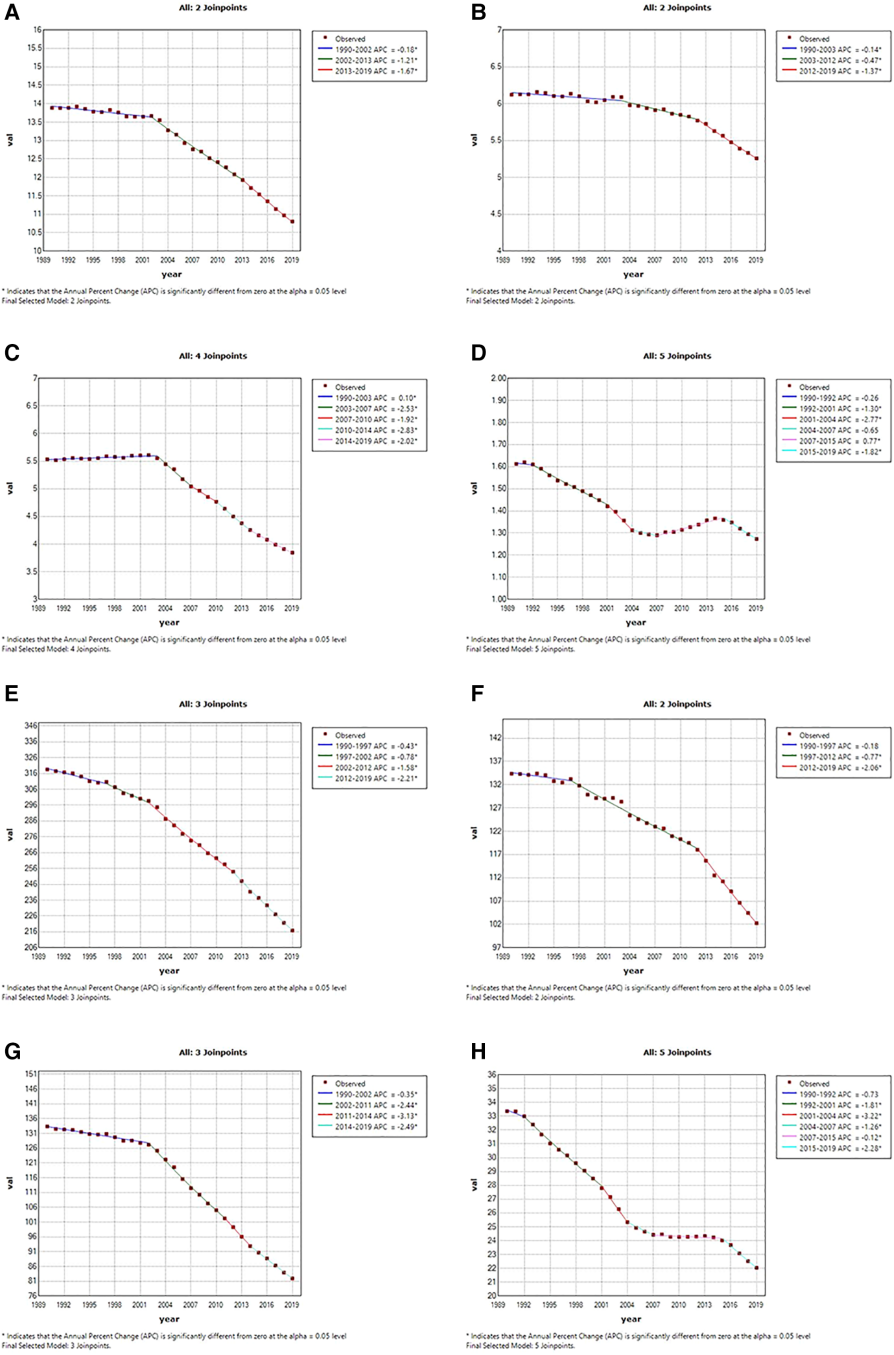
Temporal trends of global cardiovascular diseases burden attributable to lead exposure from 1990 to 2019. (**A**) Age-standardized death rates of all CVD attributable to lead exposure; (**B**) age-standardized death rates of IHD attributable to lead exposure; (**C**) age-standardized death rates of stroke attributable to lead exposure; (**D**) age-standardized death rates of HHD attributable to lead exposure. (**E**) Age-standardized DALYs rates of all CVDs attributable to lead exposure; (**F**) age-standardized DALYs rates of IHD attributable to lead exposure; (**G**) age-standardized DALYs rates of stroke attributable to lead exposure; (**H**) age-standardized DALYs rates of HHD attributable to lead exposure. CVD, cardiovascular diseases; IHD, ischemic heart disease; HHD, hypertensive heart disease.

The Joinpoint analysis was conducted to reveal the global temporal trends in CVD burden attributable to lead exposure over the past 30 years. As shown in Figure 3, the lead-attributable age-standardized deaths rate of CVD decreased slowly from 1990 to 2002 (APC = −0.18%, *p* < 0.05), followed by two significant rapid declines from 2002 to 2013 (APC = −1.21%, *p* < 0.05) and from 2013 to 2019 (APC = −1.67%, *p* < 0.05). A similar trend was observed in the age-standardized DALY rate, with the most notable decrease occurring from 2012 to 2019 (APC = −2.21%, *p* < 0.05).

For IHD, the ASR of deaths showed a steady decline from 1990 to 2012 (APCs: 1990–2003: by −0.14%, *p* < 0.05; 2003–2012: by −0.47%, *p* < 0.05), followed by a significant decline with an APC of −1.37% until 2019. Similar trends were observed in the age-standardized DALY rate of IHD attributed to lead exposure. As for Stroke, the period 2003–2007 and 2011–2014 showed the fastest decline period in the age- standardized deaths and DALYs rate attributable to lead exposure, respectively. However, we found a period of increasing trend for ASR of deaths (APC:1990–2003: by 0.10%, *p* < 0.05), also, for HHD, the age-standardized deaths rate also saw one period of significant increases attributable to lead exposure (APC:2007–2015: by 0.77%, *p* < 0.05), and had three significantly rapid declines (APC: 1992–2001: by −1.30%, *p* < 0.05; 2001–2004: by −2.77%, *p* < 0.05; 2015–2019: by −1.82%, *p* < 0.05). The age-standardized DALY rate of HHD attributable to lead exposure did not increase significantly but steadily decreased during 2007–2015.

### 3.3 Regional and national burden of cardiovascular diseases attributable to lead exposure

**Figure 4:**
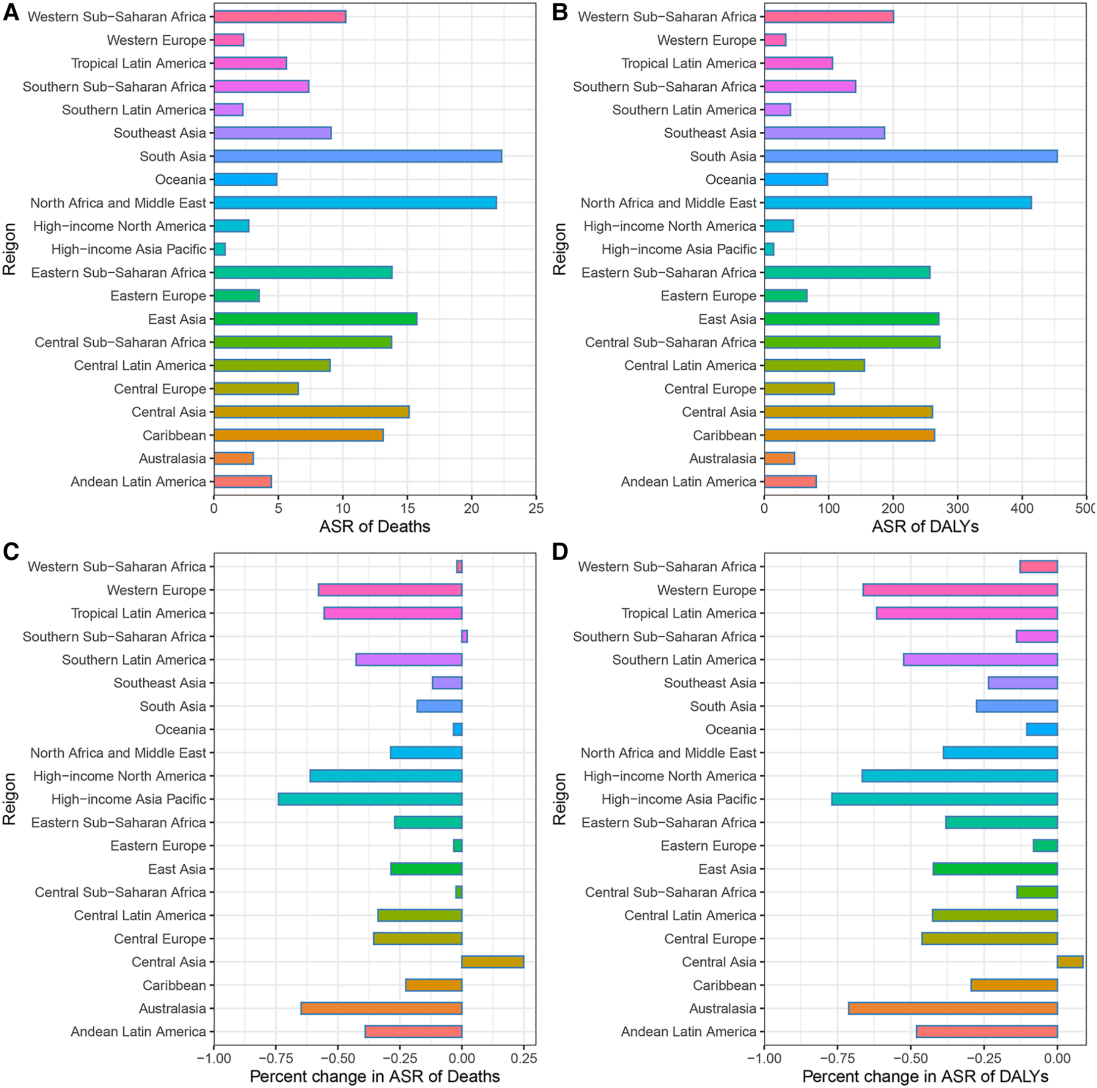
CVD burden attributable to lead exposure in 2019 and their percentage changes in rates from 1990 to 2019 across 21 GBD regions. (**A**) Age-standardized Death rates of cardiovascular diseases attributable to lead exposure in 2019. (**B**) Age-standardized DALY rates of cardiovascular diseases attributable to lead exposure in 2019. (**C**) Percentage changes in age-standardized Death rates attributable to lead exposure from 1990 to 2019. (**D**) Percentage changes in age-standardized DALY rates attributable to lead exposure from 1990 to 2019. CVD, Cardiovascular diseases; IHD, ischemic heart disease; HHD, hypertensive heart disease.

Across the 21 GBD regions in 2019, South Asia had the highest age-standardized DALY and death rates of CVD attributable to lead exposure, followed by North Africa and Middle East, while the High-income Asia Pacific had the lowest rates. High-income Asia Pacific also had the greatest decrease in age-standardized DALY and death rates from 1990 to 2019, in contrast to Central Asia and Southern Sub-Saharan Africa, which experienced significant increases in the ASR of deaths. Central Asia was the only region with a growing ASR of DALY (Figure 4; Supplementary Tables S1–S4).

**Figure 5:**
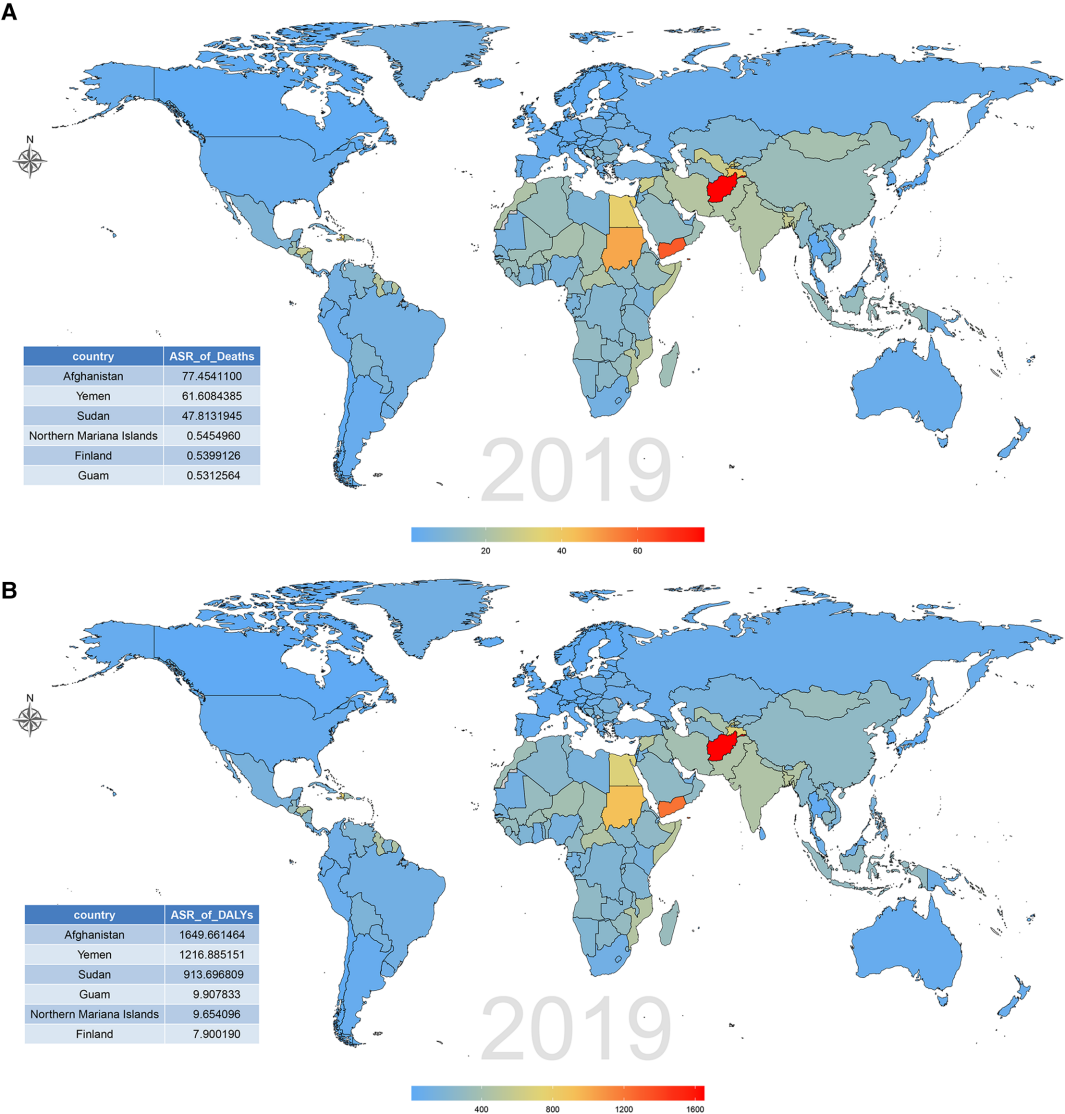
CVD burden attributable to lead exposure among 204 countries and territories in 2019. (**A**) Age-standardized death rates. (**B**) Age-standardized DALY rates. CVD, cardiovascular diseases; DALY, disability-adjusted life year.

**Figure 6:**
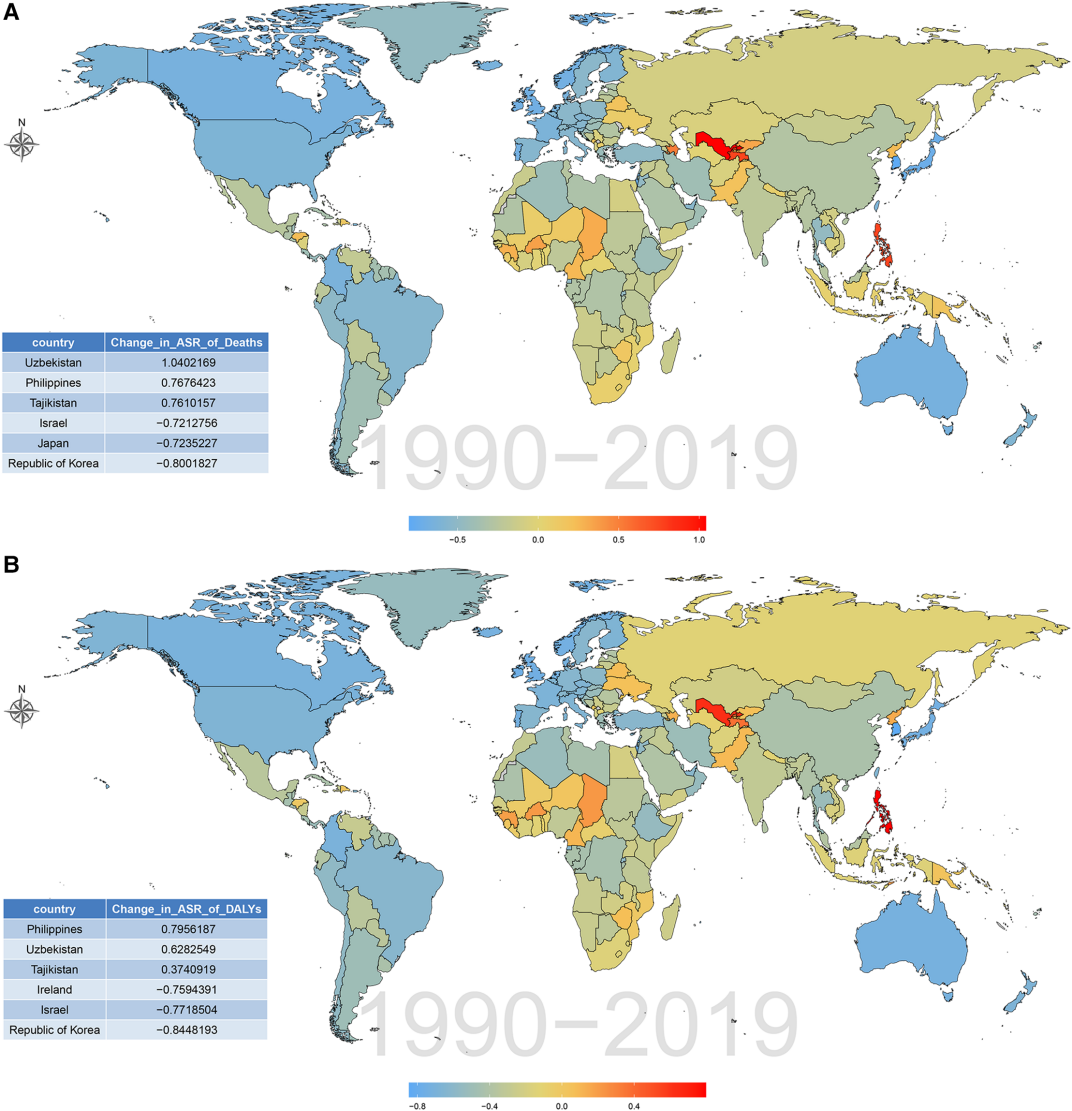
Percentage changes of CVD burden attributable to lead exposure among 204 countries and territories from 1990 to 2019. (**A**) Age-standardized death rates. (**B**) Age-standardized DALY rates. CVD, cardiovascular diseases; DALY, disability-adjusted life year.

The burden of CVD attributable to lead exposure also varied significantly between different countries and territories. In terms of ASR of deaths and DALY attributable to CVD related to lead exposure, the top three countries were Afghanistan, Yemen and Sudan. By contrast, in 2019, the countries with the lowest age-standardized death and DALY rates of CVD attributable to lead exposure was Guam and Finland, respectively (Figure 5; Supplementary Tables S5, S6). During the past 30 years, there has been considerable variation in age-standardized deaths and DALY rates of CVD attributable to lead exposure across different countries. Uzbekistan had the greatest increase in age-standardized deaths rate of CVD due to lead exposure, and Philippines showed the largest increase in age-standardized DALY rates. However, Republic of Korea showed the greatest decrease in both age-standardized deaths and DALY rates (Figure 6; Supplementary Tables S7, S8).

### 3.4 Association between SDI and CVD attributable to lead exposure

**Figure 7:**
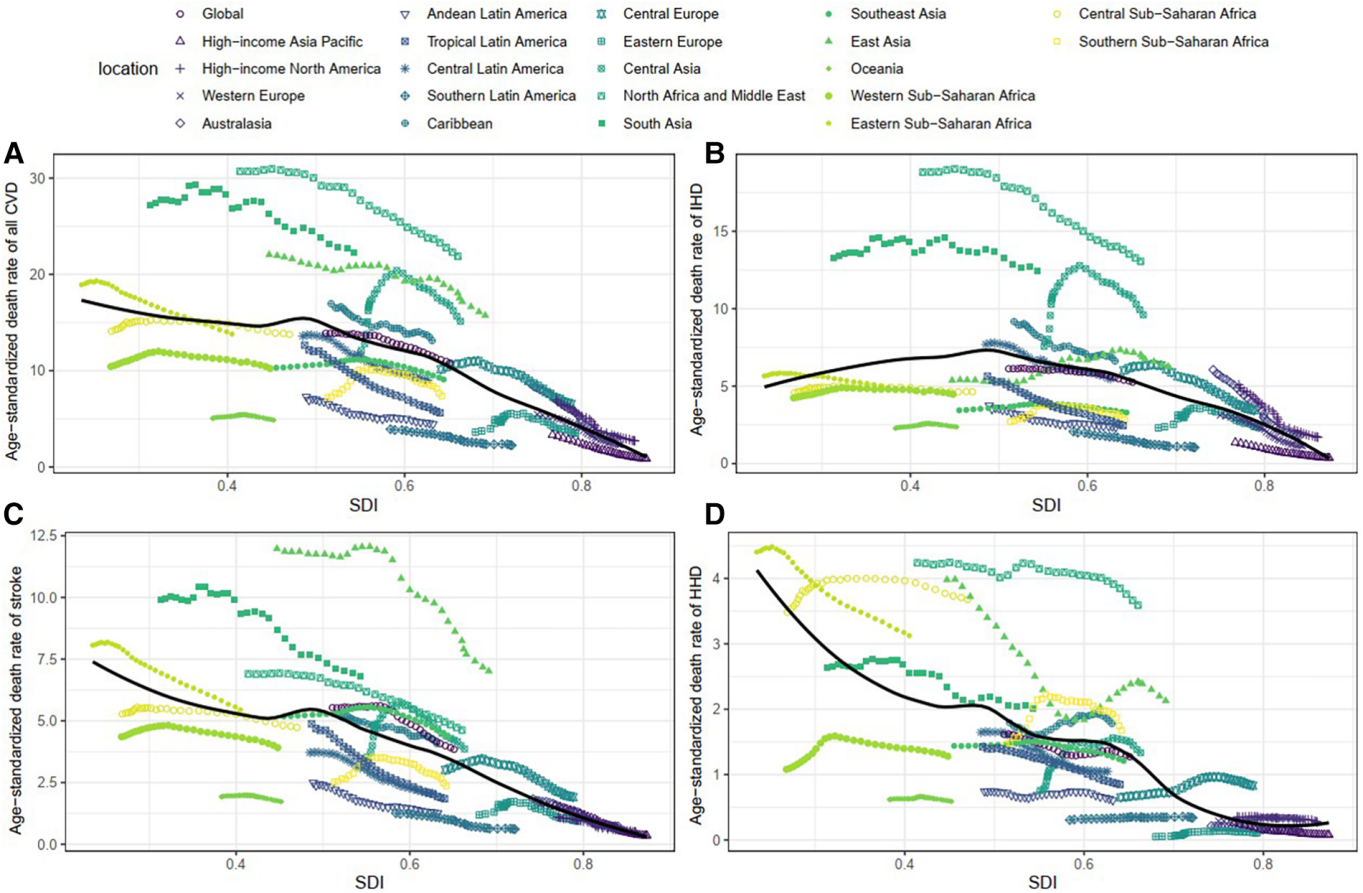
Association between age-standardized death rate and SDI among 21 regions. (**A**) Age-standardized death rates of all CVD. (**B**) Age-standardized death rates of IHD. (**C**) Age-standardized death rates of stroke. (**D**) Age-standardized death rates of HHD. IHD, ischemic heart disease. HHD, hypertensive heart disease. CVD, cardiovascular diseases; IHD, ischemic heart disease; HHD, hypertensive heart disease.

Age-standardized DALY and death rates of CVD attributable to lead exposure showed considerable variation with SDI. As shown in Figure 7; Supplementary Figure S1, countries with higher SDI experienced lower burden, as the lead-related age-standardized DALY and death rates of CVD steadily decreased with SDI. Furthermore, a similar trend was observed in the age-standardized rates of stroke and HHD and SDI. However, for IHD, the age-standardized DALY and death rates attributable to lead exposure showed a steady increase with SDI, and it peaked at the SDI of approximately 0.48, and then gradually decreased.

We observed that the low and low-middle SDI regions had the highest burden of CVD attributable to lead exposure in 2019 (Supplementary Figure S2). We also found that the lead-attributable burden of CVD has decreased among all SDI regions over the past 30 years, and high SDI region showed the largest decrease (Supplementary Figure S2).

## 4 Discussion

This study aimed to estimate the worldwide impact of chronic lead exposure on CVD and analyze its 30-year patterns. The study found that the age-standardized death and DALY rates of CVD resulting from chronic lead exposure decreased over time. We visualized the temporal trends of all CVD, IHD, Stroke and HHD from 1990 to 2019 using Joinpoint analysis. Additionally, we found the inverse association between SDI and the lead-related age-standardized DALY and death rates of CVD. In 2019, the highest burden of CVD attributable to lead exposure was observed in the regions with low and low-middle SDI regions, including South Asia, North Africa and Middle East.

Over the past 30-years, there has been a significant decrease in the burden of chronic lead exposure-related CVD at the global level. However, this trend varies by region and country. Our findings indicate that, except for Central Asia, the ASR of deaths and DALYs related to CVD have decreased in most countries and regions, with the highest reduction observed in the high-SDI region, high-income Asia Pacific. Previous studies have highlighted the significant impact of environmental factors and lifestyle on the development of CVD (16). Therefore, the decline may be primarily attributed to the implementation of lead exposure reduction strategies and the increased health consciousness among the population (17, 18). Lead was classified as the second major toxic substances by the United States of America Agency for Toxic Substances and Disease Registry (ATSDR) in 2007, and was designated as a priority heavy metal by the Ministry of Food and Drug Safety. The policy of eliminating lead from gasoline, fuels and paints in high-SDI countries has significantly reduced the amount of lead in the environment, which has led to a substantial decrease in lead levels in the population (17, 19, 20). Similarly, many developing countries, including China and Indian, have implemented laws or policies aimed at reducing lead exposure (21, 22). The widespread use of statins and low-dose aspirin for primary and secondary prevention of CVD is another contributing factor (23, 24). Furthermore, significant advancements in medical technology and increased health awareness over the past 30 years have gradually lowered the burden of CVD associated with lead exposure.

Lead exposure continues to pose a significant health challenge for low- and middle-income countries (25). Our study also found the inverse association between the SDI and burden of lead exposure-related CVD. Several factors may explain the higher age-standardized death and DALY rates of lead exposure-related CVD in low SDI regions. Apart from potential shortcomings in law enforcement, low SDI regions might have worse medical, nutritional, educational or mental healthcare conditions, which increases the burden of CVD attributed to lead exposure. Also, the aging population in these regions makes them more susceptible to CVD (26). Environmental pollution and lead exposure control measures may not receive adequate attention or implementation is another reason. A recent study in the low- and middle-income countries indicated that informal lead acid battery recycling and manufacture, metal mining and processing, electronic waste, and the use of lead as a food adulterant, primarily in spices, were major sources of lead exposure (27). Therefore, to reduce the burden of CVD associated with lead exposure in the low and middle-income regions and countries, such as Central Asia (especially in Afghanistana and Uzbekistan), North Africa and Middle East (especially in Yemen and Sudan) and South Asia, it is crucial to improve the regulation of heavy metal and electronic industries, protect specially exposed individuals, and restrict the use of lead as food adulterants. On the other hand, increasing investments in the CVD prevention and treatment are essential. Improving cardiovascular health care and increasing awareness of the risks of stroke and lead exposure may also contribute to the global decline in lead exposure-related CVD burden (28). These measures also assist in reducing the burden of other diseases associated with lead exposure, such as idiopathic developmental intellectual disability (IDII) and chronic kidney disease (20, 29).

Our results indicated that the burden of CVD attributed to lead exposure were higher among males than females. There were several possible explanations for this gender difference. First, the distribution of risk factors for CVD, such as hypertension, diabetes, obesity, alcohol drinking and smoking, are different between males and females (30). Many studies have shown that smoking and alcohol consumption increased blood lead concentrations (31, 32), and males tend to have higher blood lead levels due to their higher prevalence of smoking and alcohol consumption. Second, estrogen plays a protective role in the cardiovascular system and contributes to the sex differences observed (33). Third, a higher proportion of males work in occupations with potential lead exposure, such as construction and mechanical work, which increases their exposure to lead (34). Notably, for the elderly population, the high lead-related burden of CVD related to lead exposure may be attributed to their greater susceptibility to lead exposure and the cumulative effects of lead exposure over time (35).

Lead exposure is associated with a significant burden of specific types of CVD, with IHD and stroke being the two leading causes, followed by HHD. Several studies have found that chronic lead exposure can cause lipid disturbance by altering lipid metabolism, leading to the increased prevalence of IHD and stroke (36). Furthermore, hypertension is a known risk factor for IHD and stroke (37, 38), and previous studies have shown a positive correlation between blood lead levels and elevated systolic and diastolic blood pressure (39, 40). The IHD related to lead exposure can be explained through multiple mechanisms, including its role in elevating blood pressure by increasing vascular tension and peripheral vascular resistance (41). Lead can impair kidney function and heighten the risk of chronic kidney disease (CKD) (42). Vaziri ND et al. have shown that long-term exposure to low levels of lead can intensify the deactivation of nitric oxide by reactive oxygen species. This leads to a functional deficiency of nitric oxide and a compensatory upregulation of nitric oxide synthase, resulting in increased blood pressure due to oxidative stress and endothelial dysfunction (43). Minkyeong Kim et al. discovered that lead exposure can induce inflammation by activating the nuclear transcription factor-*κ*B, leading to cerebral vascular damage (35). Bruce P. Lanphear et al. found that lead contributes to the risk of stroke by elevating levels of oxidative stress (4, 44). Additionally, research by Marzie Boskabady et al. has identified that lead exposure increases pro-inflammatory cytokines, which in turn heightens the risk of stroke (31). Zheutlin et al. also reported that long-term lead exposure was associated with resistant hypertension (7). Therefore, chronic lead exposure significantly increases the risk of CVD, especially IHD and stroke (9).

The main advantage of our study is that it offers the first comprehensive assessment of the global burden of CVD attributable to chronic lead exposure using the latest estimated data of the GBD 2019. Although our study could raise awareness of the impact of lead exposure, there are some limitations to the GBD study. Firstly, the availability and quality of data vary in different countries, with lower SDI countries and regions potentially providing insufficient and inaccurate data, which could lead to biased results. Secondly, over the past 30 years, the GBD studies may have undergone changes in population proportions and CVD information, which is an inherent limitation of the series of GBD studies. Thirdly, in the GBD study, bone lead levels were calculated from blood lead rather than measured directly. Fourthly, GBD database did not collect data on the prevalence, awareness and treatment of lead-related CVDs. Finally, the coexistence of multiple chronic diseases is common in the older population, but the GBD study only identified one specific cause.

## 5 Conclusion

In conclusion, our study found that the elderly, males, and the low SDI regions, especially in South Asia, are at greater risk of CVD attributable to lead exposure. Furthermore, the global age-standardized deaths and DALYs rates of lead-related CVD have decreased gradually from 1999 to 2019. Based on our study results, we recommend that governments in low-SDI regions should strengthen the implementation of lead exposure prevention strategies and gradually raise awareness of self-protection among lead-exposed individuals to reduce the cardiovascular burden due to chronic lead exposure.

## Data Availability

The original contributions presented in the study are included in the article/**Supplementary Material**, further inquiries can be directed to the corresponding author.

## Supplementary material

Supplementary Figure S1 Association between age-standardized DALYs rate and SDI among 21 regions. (**A**) The age-standardized DALYs rates of all CVD. (**B**) The age-standardized DALYs rates of IHD. (**C**) The age-standardized DALYs rates of stroke. (**D**) The age-standardized DALYs rates of HHD. IHD, ischemic heart disease; HHD, hypertensive heart disease; DALY, disability-adjusted life year.

Supplementary Figure S2 CVD burden attributable to lead exposure in 2019 and their percentage changes in rates from 1990 to 2019 across different levels of SDI. (**A**) Age-standardized Death rates of CVD attributable to lead exposure in 2019. (**B**) Age-standardized DALY rates of CVD attributable to lead exposure in 2019. (**C**) Percentage changes in age-standardized death rates attributable to lead exposure from 1990 to 2019. (**D**) Percentage changes in age-standardized DALY rates attributable to lead exposure from 1990 to 2019. CVD, cardiovascular diseases; IHD, ischemic heart disease; HHD, hypertensive heart disease; DALY, disability-adjusted life year.
