# Supplementary figures and images for: Chronic lead exposure and burden of cardiovascular disease during 1990–2019: a systematic analysis of the global burden of disease study
Source: Front Cardiovasc Med. 2024 Apr 9;11:1367681. doi: 10.3389/fcvm.2024.1367681 (PMC11035890; doi:10.3389/fcvm.2024.1367681)

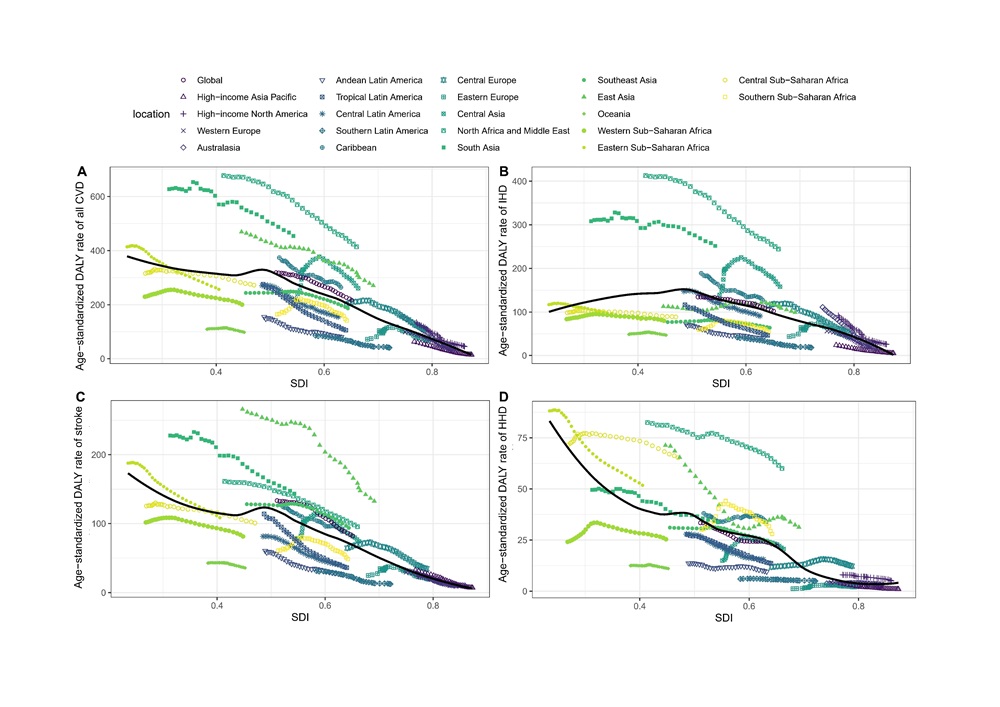

Supplement: Supplementary Figure S1 — Association between age-standardized DALYs rate and SDI among 21 regions. (A) The age-standardized DALYs rates of all CVD. (B) The age-standardized DALYs rates of IHD. (C) The age-standardized DALYs rates of stroke. (D) The age-standardized DALYs rates of HHD. IHD, ischemic heart disease; HHD, hypertensive heart disease; DALY, disability-adjusted life year. [file Datasheet1.zip › Supplementary Figure/Figure S1.jpg]

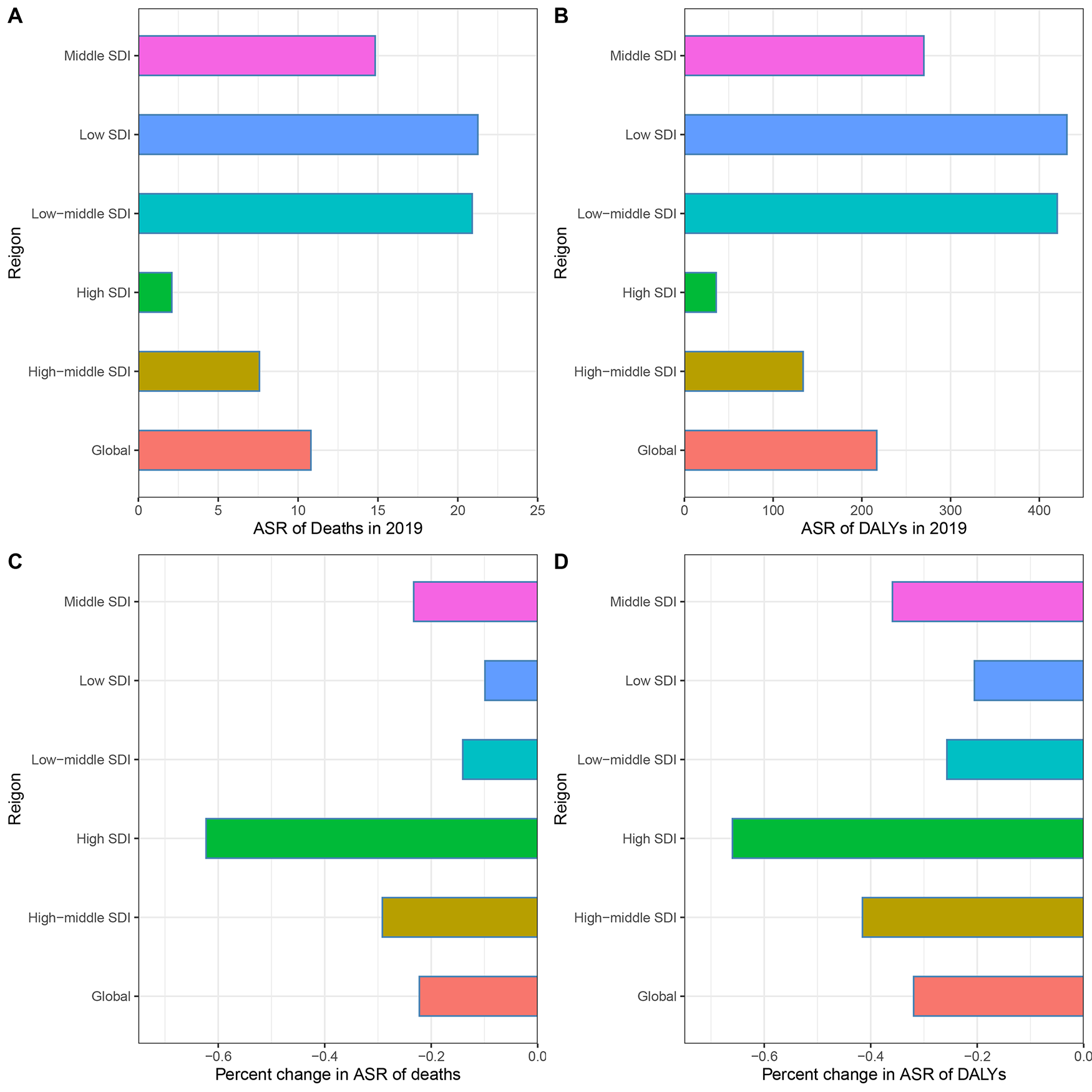

Supplement: Supplementary Figure S1 — Association between age-standardized DALYs rate and SDI among 21 regions. (A) The age-standardized DALYs rates of all CVD. (B) The age-standardized DALYs rates of IHD. (C) The age-standardized DALYs rates of stroke. (D) The age-standardized DALYs rates of HHD. IHD, ischemic heart disease; HHD, hypertensive heart disease; DALY, disability-adjusted life year. [file Datasheet1.zip › Supplementary Figure/Figure S2.tif]
