# Supplementary material for: Chronic lead exposure and burden of cardiovascular disease during 1990–2019: a systematic analysis of the global burden of disease study
Source: Front Cardiovasc Med. 2024 Apr 9;11:1367681. doi: 10.3389/fcvm.2024.1367681 (PMC11035890; doi:10.3389/fcvm.2024.1367681)
Supplement: Supplementary Figure S2 — CVD burden attributable to lead exposure in 2019 and their percentage changes in rates from 1990 to 2019 across different levels of SDI. (A) Age-standardized Death rates of CVD attributable to lead exposure in 2019. (B) Age-standardized DALY rates of CVD attributable to lead exposure in 2019. (C) Percentage changes in age-standardized death rates attributable to lead exposure from 1990 to 2019. (D) Percentage changes in age-standardized DALY rates attributable to lead exposure from 1990 to 2019. CVD, cardiovascular diseases; IHD, ischemic heart disease; HHD, hypertensive heart disease; DALY, disability-adjusted life year. [file Datasheet2.zip › Data Sheet 2_v1/Supplementary Table/Table S1.doc]

| Table S1. ASR of deaths attributable to CVD related to lead exposure in 21 regions in 2019 | | | |
| --- | --- | --- | --- |
| Regions | ASR of deaths  (per 100000 population) | 95% UI | |
|  |  | upper | lower |
| South Asia | 22.31 | 29.73 | 15.63 |
| North Africa and Middle East | 21.90 | 31.44 | 14.00 |
| East Asia | 15.74 | 22.45 | 9.69 |
| Central Asia | 15.14 | 25.72 | 5.67 |
| Eastern Sub-Saharan Africa | 13.81 | 20.51 | 8.01 |
| Central Sub-Saharan Africa | 13.78 | 22.45 | 7.02 |
| Caribbean | 13.12 | 18.72 | 8.16 |
| Western Sub-Saharan Africa | 10.23 | 15.71 | 5.43 |
| Southeast Asia | 9.09 | 14.69 | 4.25 |
| Central Latin America | 9.00 | 13.03 | 5.69 |
| Southern Sub-Saharan Africa | 7.36 | 12.59 | 3.13 |
| Central Europe | 6.54 | 11.91 | 1.98 |
| Tropical Latin America | 5.62 | 8.99 | 2.68 |
| Oceania | 4.88 | 11.66 | 0.62 |
| Andean Latin America | 4.45 | 7.15 | 2.23 |
| Eastern Europe | 3.51 | 9.69 | 0.08 |
| Australasia | 3.06 | 4.88 | 1.40 |
| High-income North America | 2.71 | 5.12 | 0.71 |
| Western Europe | 2.31 | 4.31 | 0.73 |
| Southern Latin America | 2.25 | 4.88 | 0.30 |
| High-income Asia Pacific | 0.87 | 1.97 | 0.12 |

ASR, age-standardized rates; CVD, cardiovascular diseases.
