# Supplementary material for: Chronic lead exposure and burden of cardiovascular disease during 1990–2019: a systematic analysis of the global burden of disease study
Source: Front Cardiovasc Med. 2024 Apr 9;11:1367681. doi: 10.3389/fcvm.2024.1367681 (PMC11035890; doi:10.3389/fcvm.2024.1367681)
Supplement: Supplementary Figure S2 — CVD burden attributable to lead exposure in 2019 and their percentage changes in rates from 1990 to 2019 across different levels of SDI. (A) Age-standardized Death rates of CVD attributable to lead exposure in 2019. (B) Age-standardized DALY rates of CVD attributable to lead exposure in 2019. (C) Percentage changes in age-standardized death rates attributable to lead exposure from 1990 to 2019. (D) Percentage changes in age-standardized DALY rates attributable to lead exposure from 1990 to 2019. CVD, cardiovascular diseases; IHD, ischemic heart disease; HHD, hypertensive heart disease; DALY, disability-adjusted life year. [file Datasheet2.zip › Data Sheet 2_v1/Supplementary Table/Table S2.doc]

| Table S2. ASR of DALY attributable to CVD related to lead exposure in 21 regions in 2019 | | | |
| --- | --- | --- | --- |
| Regions | ASR of DALYs  (per 100000 population) | 95% UI | |
|  |  | upper | lower |
| South Asia | 454.34 | 609.70 | 308.34 |
| North Africa and Middle East | 414.15 | 580.59 | 258.60 |
| Central Sub-Saharan Africa | 272.27 | 444.38 | 134.05 |
| East Asia | 270.79 | 385.42 | 165.75 |
| Caribbean | 264.13 | 387.94 | 158.24 |
| Central Asia | 260.80 | 464.06 | 86.52 |
| Eastern Sub-Saharan Africa | 256.82 | 387.63 | 141.84 |
| Western Sub-Saharan Africa | 200.51 | 314.18 | 101.96 |
| Southeast Asia | 186.56 | 312.00 | 80.13 |
| Central Latin America | 155.32 | 226.01 | 93.46 |
| Southern Sub-Saharan Africa | 141.79 | 241.13 | 57.38 |
| Central Europe | 108.64 | 204.54 | 28.62 |
| Tropical Latin America | 105.82 | 176.65 | 45.07 |
| Oceania | 98.02 | 256.86 | 8.50 |
| Andean Latin America | 80.65 | 134.13 | 36.47 |
| Eastern Europe | 66.24 | 190.77 | 1.14 |
| Australasia | 46.98 | 79.00 | 18.94 |
| High-income North America | 45.10 | 94.10 | 8.55 |
| Southern Latin America | 40.78 | 90.23 | 4.77 |
| Western Europe | 33.44 | 65.77 | 8.89 |
| High-income Asia Pacific | 14.62 | 36.71 | 1.43 |

ASR, age-standardized rates; CVD, cardiovascular diseases; DALY, disability-adjusted life-years.
