# Supplementary material for: Chronic lead exposure and burden of cardiovascular disease during 1990–2019: a systematic analysis of the global burden of disease study
Source: Front Cardiovasc Med. 2024 Apr 9;11:1367681. doi: 10.3389/fcvm.2024.1367681 (PMC11035890; doi:10.3389/fcvm.2024.1367681)
Supplement: Supplementary Figure S2 — CVD burden attributable to lead exposure in 2019 and their percentage changes in rates from 1990 to 2019 across different levels of SDI. (A) Age-standardized Death rates of CVD attributable to lead exposure in 2019. (B) Age-standardized DALY rates of CVD attributable to lead exposure in 2019. (C) Percentage changes in age-standardized death rates attributable to lead exposure from 1990 to 2019. (D) Percentage changes in age-standardized DALY rates attributable to lead exposure from 1990 to 2019. CVD, cardiovascular diseases; IHD, ischemic heart disease; HHD, hypertensive heart disease; DALY, disability-adjusted life year. [file Datasheet2.zip › Data Sheet 2_v1/Supplementary Table/Table S3.doc]

| Table S3. Percentage changes in ASR of deaths attributable to CVD related to lead exposure from 1990 to 2019 in 21 regions. | | | |
| --- | --- | --- | --- |
| Regions | 1990–2019, Change (%) | 95% UI | |
|  |  | upper | lower |
| Central Asia | 0.25 | 0.60 | 0.11 |
| Southern Sub-Saharan Africa | 0.02 | 0.17 | -0.10 |
| Western Sub-Saharan Africa | -0.02 | 0.24 | -0.20 |
| Central Sub-Saharan Africa | -0.02 | 0.25 | -0.20 |
| Eastern Europe | -0.03 | 9.62 | -0.15 |
| Oceania | -0.03 | 0.27 | -0.16 |
| Southeast Asia | -0.12 | 0.00 | -0.22 |
| South Asia | -0.18 | -0.07 | -0.29 |
| Caribbean | -0.23 | -0.12 | -0.32 |
| Eastern Sub-Saharan Africa | -0.27 | -0.15 | -0.37 |
| East Asia | -0.29 | -0.13 | -0.41 |
| North Africa and Middle East | -0.29 | -0.20 | -0.37 |
| Central Latin America | -0.34 | -0.25 | -0.42 |
| Central Europe | -0.36 | -0.14 | -0.45 |
| Andean Latin America | -0.39 | -0.23 | -0.50 |
| Southern Latin America | -0.43 | 0.76 | -0.50 |
| Tropical Latin America | -0.56 | -0.52 | -0.59 |
| Western Europe | -0.58 | -0.52 | -0.62 |
| High-income North America | -0.61 | -0.54 | -0.75 |
| Australasia | -0.65 | -0.62 | -0.69 |
| High-income Asia Pacific | -0.74 | -0.70 | -0.82 |

ASR, age-standardized rates; CVD, cardiovascular diseases.
