# Supplementary material for: Chronic lead exposure and burden of cardiovascular disease during 1990–2019: a systematic analysis of the global burden of disease study
Source: Front Cardiovasc Med. 2024 Apr 9;11:1367681. doi: 10.3389/fcvm.2024.1367681 (PMC11035890; doi:10.3389/fcvm.2024.1367681)
Supplement: Supplementary Figure S2 — CVD burden attributable to lead exposure in 2019 and their percentage changes in rates from 1990 to 2019 across different levels of SDI. (A) Age-standardized Death rates of CVD attributable to lead exposure in 2019. (B) Age-standardized DALY rates of CVD attributable to lead exposure in 2019. (C) Percentage changes in age-standardized death rates attributable to lead exposure from 1990 to 2019. (D) Percentage changes in age-standardized DALY rates attributable to lead exposure from 1990 to 2019. CVD, cardiovascular diseases; IHD, ischemic heart disease; HHD, hypertensive heart disease; DALY, disability-adjusted life year. [file Datasheet2.zip › Data Sheet 2_v1/Supplementary Table/Table S4.doc]

| Table S4. Percentage changes in ASR of DALY attributable to CVDs related to lead exposure from 1990 to 2019 in 21 regions. | | | |
| --- | --- | --- | --- |
| Regions | 1990–2019, Change (%) | 95% UI | |
|  |  | upper | lower |
| Central Asia | 0.09 | 0.37 | -0.03 |
| Eastern Europe | -0.08 | 8.49 | -0.19 |
| Oceania | -0.10 | 0.12 | -0.24 |
| Western Sub-Saharan Africa | -0.13 | 0.07 | -0.28 |
| Central Sub-Saharan Africa | -0.14 | 0.10 | -0.30 |
| Southern Sub-Saharan Africa | -0.14 | -0.03 | -0.25 |
| Southeast Asia | -0.23 | -0.14 | -0.33 |
| South Asia | -0.28 | -0.18 | -0.37 |
| Caribbean | -0.29 | -0.19 | -0.40 |
| Eastern Sub-Saharan Africa | -0.38 | -0.27 | -0.48 |
| North Africa and Middle East | -0.39 | -0.31 | -0.47 |
| East Asia | -0.42 | -0.30 | -0.53 |
| Central Latin America | -0.43 | -0.34 | -0.51 |
| Central Europe | -0.46 | -0.34 | -0.53 |
| Andean Latin America | -0.48 | -0.36 | -0.58 |
| Southern Latin America | -0.52 | 0.04 | -0.58 |
| Tropical Latin America | -0.62 | -0.58 | -0.67 |
| Western Europe | -0.66 | -0.62 | -0.72 |
| High-income North America | -0.67 | -0.58 | -0.83 |
| Australasia | -0.71 | -0.68 | -0.76 |
| High-income Asia Pacific | -0.77 | -0.71 | -0.87 |

ASR, age-standardized rates; CVD, cardiovascular diseases; DALY, disability-adjusted life-years.
