# Supplementary material for: Chronic lead exposure and burden of cardiovascular disease during 1990–2019: a systematic analysis of the global burden of disease study
Source: Front Cardiovasc Med. 2024 Apr 9;11:1367681. doi: 10.3389/fcvm.2024.1367681 (PMC11035890; doi:10.3389/fcvm.2024.1367681)
Supplement: Supplementary Figure S2 — CVD burden attributable to lead exposure in 2019 and their percentage changes in rates from 1990 to 2019 across different levels of SDI. (A) Age-standardized Death rates of CVD attributable to lead exposure in 2019. (B) Age-standardized DALY rates of CVD attributable to lead exposure in 2019. (C) Percentage changes in age-standardized death rates attributable to lead exposure from 1990 to 2019. (D) Percentage changes in age-standardized DALY rates attributable to lead exposure from 1990 to 2019. CVD, cardiovascular diseases; IHD, ischemic heart disease; HHD, hypertensive heart disease; DALY, disability-adjusted life year. [file Datasheet2.zip › Data Sheet 2_v1/Supplementary Table/Table S5.doc]

| Table S5. ASR of deaths attributable to CVD related to lead exposure in 204 countries and territories in 2019 | | | | |
| --- | --- | --- | --- | --- |
| Countries and territories | ASR of deaths  (per100 000 population) | 95% UI | | |
|  |  | lower | upper | |
| Afghanistan | 77.45 | 106.76 | 54.53 | |
| Yemen | 61.61 | 83.15 | 42.67 | |
| Sudan | 47.81 | 67.84 | 33.07 | |
| Tajikistan | 41.32 | 59.98 | 25.14 | |
| Haiti | 39.17 | 57.16 | 25.44 | |
| Egypt | 36.63 | 55.17 | 22.71 | |
| Syrian Arab Republic | 28.33 | 42.40 | 16.98 | |
| Honduras | 28.17 | 38.63 | 19.63 | |
| Uzbekistan | 27.45 | 46.52 | 11.33 | |
| Bangladesh | 26.39 | 35.95 | 17.49 | |
| Somalia | 24.59 | 36.83 | 14.72 | |
| Mozambique | 23.44 | 34.57 | 14.78 | |
| Guyana | 23.31 | 35.42 | 13.09 | |
| Iran (Islamic Republic of) | 22.58 | 31.09 | 15.43 | |
| India | 21.93 | 29.35 | 15.39 | |
| Pakistan | 21.42 | 30.92 | 13.36 | |
| Central African Republic | 21.28 | 34.03 | 11.15 | |
| Morocco | 20.93 | 32.16 | 11.12 | |
| Lesotho | 20.66 | 32.84 | 11.37 | |
| Solomon Islands | 20.46 | 37.68 | 6.96 | |
| Dominican Republic | 19.81 | 28.90 | 12.30 | |
| Niger | 19.34 | 27.41 | 12.44 | |
| Mongolia | 19.05 | 31.74 | 8.43 | |
| Iraq | 18.98 | 28.50 | 10.46 | |
| Nepal | 18.32 | 25.47 | 12.23 | |
| Guinea-Bissau | 18.10 | 27.59 | 10.03 | |
| Palestine | 18.10 | 27.39 | 10.00 | |
| Saint Vincent and the Grenadines | 17.91 | 25.89 | 11.16 | |
| Burkina Faso | 17.83 | 25.90 | 11.40 | |
| Chad | 17.83 | 25.93 | 11.12 | |
| Oman | 17.49 | 27.36 | 8.68 | |
| Guatemala | 17.43 | 23.87 | 11.87 | |
| Madagascar | 17.22 | 28.79 | 8.57 | |
| Mali | 16.60 | 24.05 | 10.33 | |
| Nicaragua | 16.46 | 23.26 | 10.52 | |
| China | 16.17 | 23.08 | 10.01 | |
| Azerbaijan | 16.01 | 28.34 | 5.09 | |
| Indonesia | 15.62 | 24.06 | 8.09 | |
| Algeria | 15.58 | 24.37 | 7.93 | |
| Tunisia | 15.51 | 23.92 | 8.66 | |
| Guinea | 15.42 | 23.05 | 9.09 | |
| Ethiopia | 15.25 | 21.93 | 9.56 | |
| Zimbabwe | 15.03 | 22.72 | 8.42 | |
| Timor-Leste | 15.02 | 23.40 | 7.68 | |
| Burundi | 14.30 | 22.10 | 7.65 | |
| Lao People's Democratic Republic | 14.09 | 22.36 | 6.82 | |
| Saudi Arabia | 14.08 | 21.15 | 7.81 | |
| Angola | 14.04 | 21.84 | 7.59 | |
| Cambodia | 13.95 | 20.99 | 7.62 | |
| Equatorial Guinea | 13.74 | 21.29 | 7.68 | |
| Democratic Republic of the Congo | 13.73 | 22.70 | 6.86 | |
| Gambia | 13.71 | 21.36 | 7.50 | |
| Eritrea | 13.63 | 22.91 | 6.61 | |
| Benin | 13.51 | 20.62 | 7.70 | |
| Kyrgyzstan | 13.42 | 22.30 | 5.32 | |
| Malawi | 13.36 | 20.43 | 7.51 | |
| Bosnia and Herzegovina | 13.30 | 20.76 | 7.21 | |
| Cameroon | 12.87 | 20.23 | 7.07 | |
| Sierra Leone | 12.71 | 20.31 | 6.92 | |
| North Macedonia | 12.52 | 22.82 | 3.62 | |
| Liberia | 12.51 | 19.29 | 6.93 | |
| Myanmar | 12.48 | 19.97 | 5.98 | |
| Botswana | 12.38 | 20.25 | 5.88 | |
| South Sudan | 12.27 | 19.33 | 6.70 | |
| Zambia | 12.15 | 20.44 | 5.46 | |
| Lebanon | 12.10 | 19.20 | 5.77 | |
| Bulgaria | 12.08 | 23.82 | 3.01 | |
| El Salvador | 12.05 | 17.48 | 7.74 | |
| Democratic People's Republic of Korea | 12.02 | 19.32 | 5.62 | |
| Turkmenistan | 11.94 | 22.57 | 3.20 | |
| Albania | 11.88 | 19.45 | 6.14 | |
| Grenada | 11.74 | 17.79 | 6.60 | |
| Uganda | 11.32 | 17.73 | 6.12 | |
| Eswatini | 11.26 | 19.98 | 4.55 | |
| United Republic of Tanzania | 11.12 | 17.48 | 5.66 | |
| Kiribati | 10.90 | 21.89 | 2.77 | |
| Bhutan | 10.54 | 16.38 | 5.80 | |
| Venezuela (Bolivarian Republic of) | 10.53 | 16.55 | 5.69 | |
| Congo | 10.51 | 19.09 | 3.80 | |
| Namibia | 10.49 | 17.25 | 4.48 | |
| Suriname | 10.48 | 16.16 | 5.55 | |
| Rwanda | 10.34 | 16.59 | 5.13 | |
| Djibouti | 10.27 | 17.06 | 4.80 | |
| Serbia | 10.00 | 17.89 | 3.30 | |
| Sao Tome and Principe | 9.97 | 15.90 | 4.40 | |
| Togo | 9.96 | 16.62 | 4.57 | |
| Bolivia (Plurinational State of) | 9.83 | 15.60 | 5.32 | |
| Comoros | 9.82 | 16.30 | 4.58 | |
| Côte d'Ivoire | 9.74 | 15.62 | 4.61 | |
| Armenia | 9.59 | 16.43 | 3.73 | |
| Micronesia (Federated States of) | 9.55 | 20.35 | 2.49 | |
| Georgia | 9.54 | 19.36 | 2.11 | |
| Cuba | 9.42 | 14.12 | 5.45 | |
| Romania | 9.31 | 16.59 | 3.13 | |
| Jordan | 9.06 | 15.31 | 4.06 | |
| Malta | 9.06 | 12.78 | 6.03 | |
| Senegal | 9.04 | 14.69 | 4.19 | |
| Kazakhstan | 8.99 | 17.20 | 2.08 | |
| Vanuatu | 8.97 | 19.47 | 1.55 | |
| Jamaica | 8.80 | 13.82 | 4.69 | |
| Marshall Islands | 8.76 | 18.64 | 1.74 | |
| Libya | 8.75 | 15.16 | 3.67 | |
| Mexico | 8.59 | 12.35 | 5.34 | |
| Viet Nam | 8.57 | 14.58 | 3.36 | |
| Saint Lucia | 8.46 | 13.37 | 4.31 | |
| Dominica | 8.32 | 14.79 | 3.39 | |
| Kenya | 8.23 | 13.70 | 3.72 | |
| Saint Kitts and Nevis | 7.89 | 13.49 | 3.07 | |
| Paraguay | 7.68 | 12.27 | 3.82 | |
| United Arab Emirates | 7.47 | 15.59 | 1.88 | |
| Gabon | 7.40 | 14.24 | 2.08 | |
| Nigeria | 7.38 | 12.33 | 3.23 | |
| Philippines | 7.13 | 13.78 | 2.88 | |
| Qatar | 7.10 | 13.35 | 1.96 | |
| Costa Rica | 6.80 | 10.39 | 3.95 | |
| Ghana | 6.69 | 12.70 | 1.79 | |
| Tokelau | 6.67 | 12.43 | 2.87 | |
| Republic of Moldova | 6.56 | 13.85 | 0.96 | |
| Belize | 6.53 | 10.51 | 3.07 | |
| Mauritania | 6.40 | 11.09 | 2.45 | |
| Maldives | 6.36 | 10.61 | 2.84 | |
| Kuwait | 6.24 | 10.33 | 2.74 | |
| Cabo Verde | 6.10 | 10.85 | 1.97 | |
| Antigua and Barbuda | 6.08 | 10.87 | 2.21 | |
| Bahrain | 6.01 | 10.78 | 2.21 | |
| Belarus | 5.95 | 13.72 | 0.57 | |
| Panama | 5.88 | 8.83 | 3.32 | |
| Colombia | 5.79 | 9.23 | 3.17 | |
| Greenland | 5.76 | 9.80 | 2.33 | |
| South Africa | 5.68 | 10.43 | 1.93 | |
| Brazil | 5.57 | 8.90 | 2.64 | |
| Turkey | 5.35 | 9.83 | 1.74 | |
| Montenegro | 5.30 | 12.10 | 0.17 | |
| Portugal | 5.02 | 7.36 | 2.86 | |
| Poland | 4.95 | 9.13 | 1.51 | |
| Ecuador | 4.79 | 8.26 | 1.89 | |
| Brunei Darussalam | 4.77 | 9.36 | 1.12 | |
| Cyprus | 4.77 | 8.20 | 1.84 | |
| Papua New Guinea | 4.68 | 11.52 | 0.37 | |
| Malaysia | 4.63 | 9.32 | 0.78 | |
| Belgium | 4.58 | 6.60 | 2.65 | |
| Bahamas | 4.53 | 9.89 | 0.78 | |
| Slovakia | 4.50 | 9.70 | 0.52 | |
| Uruguay | 4.46 | 7.35 | 1.73 | |
| United States Virgin Islands | 4.38 | 9.42 | 0.53 | |
| Tuvalu | 4.30 | 11.29 | 0.45 | |
| Hungary | 4.28 | 9.55 | 0.53 | |
| Greece | 4.13 | 7.25 | 1.43 | |
| Ukraine | 4.10 | 12.17 | 0.02 | |
| Barbados | 3.95 | 7.20 | 1.19 | |
| Nauru | 3.79 | 11.99 | 0.26 | |
| Croatia | 3.69 | 8.13 | 0.22 | |
| Seychelles | 3.45 | 8.40 | 0.19 | |
| Czechia | 3.44 | 7.29 | 0.39 | |
| Samoa | 3.24 | 9.19 | 0.07 | |
| Mauritius | 3.20 | 7.26 | 0.43 | |
| Peru | 3.17 | 5.32 | 1.56 | |
| Russian Federation | 3.15 | 8.87 | 0.04 | |
| Australia | 3.07 | 4.84 | 1.46 | |
| New Zealand | 2.98 | 5.19 | 1.03 | |
| Spain | 2.93 | 4.79 | 1.30 | |
| United States of America | 2.92 | 5.45 | 0.79 | |
| Italy | 2.88 | 5.36 | 0.96 | |
| Sri Lanka | 2.73 | 6.46 | 0.15 | |
| Argentina | 2.65 | 5.70 | 0.29 | |
| Ireland | 2.65 | 4.73 | 0.80 | |
| Taiwan (Province of China) | 2.61 | 4.74 | 0.87 | |
| Bermuda | 2.54 | 5.08 | 0.55 | |
| Latvia | 2.51 | 7.19 | 0.00 | |
| Fiji | 2.38 | 8.01 | 0.00 | |
| Trinidad and Tobago | 2.34 | 6.00 | 0.11 | |
| France | 2.21 | 3.68 | 0.91 | |
| Singapore | 2.13 | 3.90 | 0.63 | |
| Republic of Korea | 2.10 | 3.87 | 0.67 | |
| Lithuania | 2.02 | 6.45 | 0.00 | |
| Monaco | 1.98 | 4.09 | 0.29 | |
| Slovenia | 1.98 | 4.51 | 0.14 | |
| Austria | 1.90 | 4.34 | 0.25 | |
| San Marino | 1.88 | 3.94 | 0.33 | |
| Tonga | 1.80 | 5.07 | 0.03 | |
| Germany | 1.78 | 4.27 | 0.10 | |
| Estonia | 1.73 | 6.08 | 0.00 | |
| Niue | 1.64 | 4.87 | 0.47 | |
| Puerto Rico | 1.58 | 3.52 | 0.16 | |
| Norway | 1.58 | 3.17 | 0.30 | |
| Netherlands | 1.56 | 3.12 | 0.24 | |
| Switzerland | 1.54 | 3.26 | 0.16 | |
| Andorra | 1.52 | 3.34 | 0.18 | |
| United Kingdom | 1.50 | 3.29 | 0.23 | |
| Luxembourg | 1.48 | 3.22 | 0.13 | |
| Iceland | 1.47 | 3.19 | 0.14 | |
| Israel | 1.38 | 2.79 | 0.21 | |
| Denmark | 1.28 | 2.94 | 0.01 | |
| Thailand | 1.27 | 3.29 | 0.04 | |
| American Samoa | 1.03 | 4.14 | 0.00 | |
| Sweden | 1.02 | 2.81 | 0.01 | |
| Canada | 0.97 | 2.44 | 0.01 | |
| Palau | 0.87 | 4.10 | 0.04 | |
| Chile | 0.78 | 2.49 | 0.00 | |
| Cook Islands | 0.70 | 3.14 | 0.04 | |
| Japan | 0.64 | 1.64 | 0.02 | |
| Northern Mariana Islands | 0.55 | 2.92 | 0.00 | |
| Finland | 0.54 | 2.29 | 0.00 | |
| Guam | 0.53 | 2.72 | 0.00 | |

ASR, age-standardized rates; CVD, cardiovascular diseases.
