# Supplementary material for: Chronic lead exposure and burden of cardiovascular disease during 1990–2019: a systematic analysis of the global burden of disease study
Source: Front Cardiovasc Med. 2024 Apr 9;11:1367681. doi: 10.3389/fcvm.2024.1367681 (PMC11035890; doi:10.3389/fcvm.2024.1367681)
Supplement: Supplementary Figure S2 — CVD burden attributable to lead exposure in 2019 and their percentage changes in rates from 1990 to 2019 across different levels of SDI. (A) Age-standardized Death rates of CVD attributable to lead exposure in 2019. (B) Age-standardized DALY rates of CVD attributable to lead exposure in 2019. (C) Percentage changes in age-standardized death rates attributable to lead exposure from 1990 to 2019. (D) Percentage changes in age-standardized DALY rates attributable to lead exposure from 1990 to 2019. CVD, cardiovascular diseases; IHD, ischemic heart disease; HHD, hypertensive heart disease; DALY, disability-adjusted life year. [file Datasheet2.zip › Data Sheet 2_v1/Supplementary Table/Table S6.doc]

| Table S6. ASR of DALY attributable to CVD related to lead exposure in 204 countries and territories in 2019 | | | |
| --- | --- | --- | --- |
| Countries and territories | ASR of DALYs  (per100 000 population) | 95% UI | |
|  |  | lower | upper |
| Afghanistan | 1649.66 | 2226.39 | 1155.88 |
| Yemen | 1216.89 | 1666.49 | 848.29 |
| Sudan | 913.70 | 1296.40 | 624.62 |
| Haiti | 763.27 | 1134.13 | 474.71 |
| Egypt | 698.35 | 1060.08 | 418.53 |
| Tajikistan | 687.25 | 1007.18 | 406.52 |
| Bangladesh | 504.72 | 692.02 | 332.44 |
| Somalia | 502.97 | 757.77 | 292.83 |
| Honduras | 490.59 | 676.62 | 338.49 |
| Syrian Arab Republic | 490.13 | 745.18 | 280.24 |
| Mozambique | 472.21 | 698.04 | 285.25 |
| Central African Republic | 457.85 | 734.92 | 229.39 |
| India | 450.41 | 606.09 | 305.82 |
| Guyana | 449.22 | 705.52 | 239.65 |
| Pakistan | 446.71 | 664.03 | 264.27 |
| Solomon Islands | 437.67 | 891.42 | 114.77 |
| Lesotho | 435.28 | 695.43 | 234.46 |
| Uzbekistan | 417.85 | 755.16 | 145.11 |
| Iran (Islamic Republic of) | 391.80 | 528.62 | 270.77 |
| Niger | 389.10 | 568.50 | 244.31 |
| Guinea-Bissau | 379.91 | 600.91 | 197.82 |
| Dominican Republic | 372.15 | 556.74 | 216.27 |
| Chad | 367.70 | 543.73 | 222.11 |
| Morocco | 361.15 | 564.27 | 177.52 |
| Burkina Faso | 358.88 | 528.39 | 221.41 |
| Nepal | 358.48 | 497.78 | 234.94 |
| Iraq | 348.52 | 538.59 | 177.06 |
| Mongolia | 334.10 | 594.16 | 124.71 |
| Madagascar | 325.07 | 566.89 | 142.19 |
| Mali | 313.12 | 461.11 | 186.52 |
| Guinea | 312.31 | 478.19 | 172.62 |
| Zimbabwe | 305.44 | 471.64 | 166.52 |
| Indonesia | 300.89 | 479.54 | 145.80 |
| Palestine | 292.32 | 449.80 | 154.36 |
| Saint Vincent and the Grenadines | 292.19 | 427.88 | 172.73 |
| Timor-Leste | 291.83 | 468.74 | 131.65 |
| Guatemala | 284.79 | 390.11 | 194.72 |
| Lao People's Democratic Republic | 283.58 | 470.74 | 127.58 |
| Cambodia | 281.76 | 436.51 | 146.23 |
| China | 276.26 | 392.77 | 170.20 |
| Angola | 275.01 | 437.01 | 141.51 |
| Saudi Arabia | 274.66 | 428.68 | 139.48 |
| Oman | 273.95 | 440.27 | 123.06 |
| Ethiopia | 272.23 | 394.12 | 167.01 |
| Democratic Republic of the Congo | 271.05 | 447.66 | 128.89 |
| Burundi | 269.04 | 434.47 | 130.81 |
| Cameroon | 268.85 | 430.71 | 140.61 |
| Benin | 264.39 | 407.20 | 143.37 |
| Gambia | 262.96 | 425.92 | 133.37 |
| Tunisia | 256.88 | 407.42 | 133.47 |
| Malawi | 254.12 | 393.85 | 136.88 |
| Sierra Leone | 253.26 | 413.87 | 128.58 |
| Eritrea | 251.63 | 432.88 | 110.86 |
| Kiribati | 248.85 | 543.19 | 45.87 |
| Myanmar | 248.26 | 413.84 | 111.61 |
| Democratic People's Republic of Korea | 247.91 | 414.95 | 105.04 |
| Liberia | 244.57 | 391.22 | 128.47 |
| Azerbaijan | 241.94 | 450.07 | 62.53 |
| Botswana | 241.47 | 404.55 | 107.43 |
| Equatorial Guinea | 240.91 | 382.09 | 126.72 |
| Nicaragua | 238.27 | 339.34 | 149.70 |
| Algeria | 231.64 | 373.10 | 109.41 |
| Kyrgyzstan | 227.57 | 394.33 | 78.64 |
| Eswatini | 226.62 | 406.23 | 83.30 |
| South Sudan | 225.39 | 363.70 | 114.93 |
| Zambia | 217.76 | 380.46 | 85.36 |
| Turkmenistan | 217.42 | 434.24 | 43.47 |
| Bosnia and Herzegovina | 215.62 | 344.05 | 110.28 |
| El Salvador | 214.32 | 311.15 | 137.14 |
| Uganda | 211.95 | 335.83 | 108.25 |
| Grenada | 207.33 | 319.31 | 107.05 |
| Micronesia (Federated States of) | 205.29 | 473.01 | 41.02 |
| Namibia | 199.77 | 337.36 | 82.07 |
| United Republic of Tanzania | 199.47 | 320.04 | 92.52 |
| Suriname | 199.13 | 324.06 | 93.02 |
| Venezuela (Bolivarian Republic of) | 197.98 | 320.25 | 102.05 |
| Bulgaria | 197.60 | 411.00 | 38.40 |
| Lebanon | 196.57 | 327.54 | 85.00 |
| Togo | 194.32 | 341.28 | 77.91 |
| Congo | 193.65 | 363.62 | 60.70 |
| Albania | 188.35 | 317.02 | 89.55 |
| Côte d'Ivoire | 187.95 | 315.71 | 78.78 |
| Bhutan | 187.40 | 302.50 | 93.33 |
| North Macedonia | 186.74 | 355.64 | 46.15 |
| Djibouti | 182.89 | 317.47 | 76.00 |
| Sao Tome and Principe | 182.24 | 311.05 | 70.56 |
| Rwanda | 181.98 | 307.40 | 82.37 |
| Vanuatu | 175.88 | 432.26 | 18.25 |
| Bolivia (Plurinational State of) | 175.51 | 284.32 | 90.80 |
| Comoros | 169.71 | 294.84 | 68.10 |
| Viet Nam | 167.95 | 293.48 | 59.97 |
| Cuba | 167.54 | 256.33 | 91.67 |
| Marshall Islands | 166.60 | 407.45 | 21.03 |
| Senegal | 164.91 | 280.01 | 67.16 |
| Georgia | 162.35 | 337.48 | 28.68 |
| Philippines | 157.02 | 304.10 | 60.81 |
| Malta | 156.63 | 215.58 | 105.93 |
| Jamaica | 156.03 | 256.22 | 75.58 |
| Romania | 154.29 | 287.36 | 45.16 |
| Armenia | 152.82 | 268.50 | 52.05 |
| Jordan | 152.09 | 258.87 | 61.67 |
| Serbia | 151.10 | 278.78 | 44.71 |
| Libya | 150.89 | 276.29 | 53.95 |
| Kazakhstan | 146.58 | 300.50 | 25.83 |
| Kenya | 146.37 | 253.20 | 60.14 |
| Mexico | 144.32 | 214.75 | 85.11 |
| Nigeria | 142.12 | 242.46 | 60.16 |
| Saint Lucia | 137.07 | 226.56 | 61.51 |
| Paraguay | 136.12 | 227.38 | 61.74 |
| Gabon | 133.28 | 266.58 | 32.14 |
| Dominica | 131.02 | 246.95 | 45.25 |
| Saint Kitts and Nevis | 130.94 | 240.73 | 41.23 |
| United Arab Emirates | 124.15 | 266.21 | 21.94 |
| Ghana | 122.59 | 248.95 | 25.63 |
| Republic of Moldova | 122.06 | 259.54 | 15.26 |
| Costa Rica | 119.39 | 183.33 | 65.34 |
| Belize | 116.42 | 195.61 | 46.93 |
| Mauritania | 115.37 | 212.36 | 36.62 |
| Belarus | 114.83 | 266.14 | 8.86 |
| Maldives | 113.70 | 193.24 | 45.72 |
| Kuwait | 113.57 | 197.11 | 44.36 |
| Greenland | 105.18 | 187.20 | 36.04 |
| Brazil | 105.12 | 175.77 | 44.61 |
| Cabo Verde | 103.55 | 192.64 | 26.75 |
| South Africa | 103.41 | 193.28 | 30.79 |
| Panama | 102.10 | 159.63 | 54.52 |
| Qatar | 99.10 | 197.05 | 22.40 |
| Antigua and Barbuda | 95.86 | 180.50 | 27.51 |
| Bahrain | 93.72 | 174.85 | 29.32 |
| Malaysia | 92.98 | 196.41 | 12.46 |
| Colombia | 90.81 | 149.68 | 45.51 |
| Papua New Guinea | 89.16 | 248.23 | 3.60 |
| Uruguay | 85.80 | 143.20 | 31.48 |
| Poland | 84.69 | 158.23 | 22.91 |
| Turkey | 83.88 | 164.68 | 21.05 |
| Montenegro | 83.06 | 199.22 | 1.30 |
| Bahamas | 77.28 | 184.76 | 8.21 |
| Tuvalu | 77.28 | 241.62 | 3.16 |
| Portugal | 76.89 | 115.49 | 39.88 |
| Tokelau | 74.90 | 189.87 | 20.16 |
| Ecuador | 74.88 | 138.33 | 25.30 |
| Hungary | 74.33 | 169.59 | 6.84 |
| Brunei Darussalam | 73.90 | 157.24 | 13.88 |
| Belgium | 72.05 | 107.67 | 38.24 |
| Slovakia | 71.62 | 162.35 | 5.96 |
| Ukraine | 70.91 | 225.17 | 0.17 |
| Nauru | 69.50 | 263.27 | 1.61 |
| United States Virgin Islands | 67.95 | 158.41 | 4.96 |
| Greece | 65.52 | 119.98 | 19.34 |
| Seychelles | 65.12 | 162.28 | 2.05 |
| Cyprus | 65.03 | 115.84 | 21.19 |
| Barbados | 63.28 | 125.64 | 14.83 |
| Russian Federation | 61.42 | 176.59 | 0.60 |
| Mauritius | 61.37 | 146.27 | 5.82 |
| Samoa | 59.93 | 190.15 | 0.48 |
| Peru | 59.24 | 103.21 | 26.01 |
| Croatia | 53.79 | 126.96 | 1.68 |
| Czechia | 53.08 | 119.81 | 4.34 |
| Taiwan (Province of China) | 50.95 | 97.15 | 14.19 |
| Sri Lanka | 50.12 | 126.32 | 1.59 |
| New Zealand | 49.65 | 88.22 | 17.03 |
| Argentina | 48.76 | 106.93 | 4.22 |
| United States of America | 48.67 | 100.47 | 9.61 |
| Australia | 46.45 | 77.63 | 19.16 |
| Latvia | 45.87 | 135.65 | 0.00 |
| Spain | 44.35 | 75.07 | 17.09 |
| Fiji | 43.02 | 162.88 | 0.00 |
| Trinidad and Tobago | 39.60 | 113.77 | 0.95 |
| Bermuda | 38.67 | 83.72 | 5.93 |
| Ireland | 38.56 | 74.31 | 9.45 |
| Italy | 38.02 | 73.13 | 10.30 |
| Singapore | 36.87 | 71.91 | 8.08 |
| Lithuania | 36.10 | 116.77 | 0.00 |
| Tonga | 33.05 | 104.22 | 0.18 |
| France | 32.74 | 58.53 | 11.06 |
| Republic of Korea | 31.04 | 59.70 | 7.57 |
| Slovenia | 29.87 | 70.75 | 1.44 |
| Estonia | 29.66 | 102.98 | 0.00 |
| Monaco | 28.68 | 64.66 | 3.03 |
| San Marino | 27.17 | 63.08 | 3.51 |
| Puerto Rico | 26.83 | 67.98 | 1.56 |
| Thailand | 26.68 | 73.53 | 0.41 |
| Germany | 25.97 | 64.61 | 0.89 |
| Norway | 24.15 | 51.98 | 3.78 |
| Austria | 24.02 | 58.78 | 1.92 |
| United Kingdom | 22.79 | 54.35 | 2.73 |
| Netherlands | 22.50 | 48.90 | 2.37 |
| Andorra | 22.44 | 52.11 | 1.88 |
| Luxembourg | 21.47 | 50.10 | 1.19 |
| Switzerland | 20.91 | 46.36 | 1.63 |
| Iceland | 20.58 | 48.38 | 1.14 |
| Israel | 19.80 | 43.75 | 2.00 |
| Niue | 19.34 | 88.64 | 2.95 |
| American Samoa | 18.96 | 88.98 | 0.01 |
| Denmark | 18.84 | 46.85 | 0.04 |
| Canada | 15.93 | 43.68 | 0.06 |
| Palau | 15.81 | 88.58 | 0.20 |
| Sweden | 15.56 | 44.96 | 0.13 |
| Chile | 13.14 | 44.26 | 0.00 |
| Cook Islands | 11.16 | 62.70 | 0.21 |
| Japan | 10.21 | 30.21 | 0.24 |
| Guam | 9.91 | 58.83 | 0.00 |
| Northern Mariana Islands | 9.65 | 57.05 | 0.00 |
| Finland | 7.90 | 35.49 | 0.00 |

ASR, age-standardized rates; CVD, cardiovascular diseases; DALY, disability-adjusted life-years.
