# Supplementary material for: Chronic lead exposure and burden of cardiovascular disease during 1990–2019: a systematic analysis of the global burden of disease study
Source: Front Cardiovasc Med. 2024 Apr 9;11:1367681. doi: 10.3389/fcvm.2024.1367681 (PMC11035890; doi:10.3389/fcvm.2024.1367681)
Supplement: Supplementary Figure S2 — CVD burden attributable to lead exposure in 2019 and their percentage changes in rates from 1990 to 2019 across different levels of SDI. (A) Age-standardized Death rates of CVD attributable to lead exposure in 2019. (B) Age-standardized DALY rates of CVD attributable to lead exposure in 2019. (C) Percentage changes in age-standardized death rates attributable to lead exposure from 1990 to 2019. (D) Percentage changes in age-standardized DALY rates attributable to lead exposure from 1990 to 2019. CVD, cardiovascular diseases; IHD, ischemic heart disease; HHD, hypertensive heart disease; DALY, disability-adjusted life year. [file Datasheet2.zip › Data Sheet 2_v1/Supplementary Table/Table S8.doc]

| Table S8. Percentage changes in ASR of DALY attributable to CVD related to lead exposure from 1990 to 2019 in 204 countries and territories. | | | |
| --- | --- | --- | --- |
| Countries and territories | 1990–2019, Change (%) | 95% UI | |
|  |  | lower | upper |
| Philippines | 0.80 | 1.50 | 0.20 |
| Uzbekistan | 0.63 | 1.03 | 0.40 |
| Tajikistan | 0.37 | 0.69 | 0.12 |
| Timor-Leste | 0.24 | 0.59 | -0.06 |
| Lesotho | 0.23 | 0.63 | -0.08 |
| Chad | 0.22 | 0.56 | -0.02 |
| Burkina Faso | 0.22 | 0.54 | -0.04 |
| Guinea | 0.19 | 0.57 | -0.08 |
| Democratic People's Republic of Korea | 0.15 | 0.67 | -0.10 |
| Azerbaijan | 0.12 | 0.75 | -0.05 |
| Cameroon | 0.11 | 0.50 | -0.16 |
| Pakistan | 0.09 | 0.36 | -0.11 |
| Belarus | 0.07 | 83.80 | -0.16 |
| Zimbabwe | 0.07 | 0.33 | -0.15 |
| Kyrgyzstan | 0.06 | 0.67 | -0.12 |
| Gambia | 0.06 | 0.43 | -0.22 |
| Ukraine | 0.06 | 68.47 | -0.20 |
| Montenegro | 0.04 | 59.23 | -0.15 |
| Papua New Guinea | 0.04 | 4.65 | -0.19 |
| Sao Tome and Principe | 0.03 | 0.29 | -0.18 |
| Dominican Republic | 0.03 | 0.33 | -0.22 |
| Niger | 0.02 | 0.33 | -0.19 |
| Sierra Leone | 0.01 | 0.34 | -0.20 |
| Honduras | -0.01 | 0.23 | -0.19 |
| Republic of Moldova | -0.01 | 50.84 | -0.22 |
| Mozambique | -0.01 | 0.26 | -0.24 |
| Liberia | -0.03 | 0.32 | -0.26 |
| Mali | -0.04 | 0.21 | -0.23 |
| Democratic Republic of the Congo | -0.05 | 0.30 | -0.26 |
| Solomon Islands | -0.07 | 0.20 | -0.30 |
| Togo | -0.08 | 0.19 | -0.25 |
| Central African Republic | -0.08 | 0.20 | -0.29 |
| Turkmenistan | -0.08 | 0.33 | -0.26 |
| Benin | -0.09 | 0.17 | -0.26 |
| Vanuatu | -0.09 | 0.21 | -0.43 |
| Guinea-Bissau | -0.09 | 0.23 | -0.34 |
| Nepal | -0.12 | 0.14 | -0.33 |
| North Macedonia | -0.12 | 0.45 | -0.30 |
| Ghana | -0.13 | 0.19 | -0.32 |
| Russian Federation | -0.13 | 5.71 | -0.27 |
| Cabo Verde | -0.14 | 0.00 | -0.29 |
| Kiribati | -0.15 | 0.32 | -0.34 |
| Indonesia | -0.15 | -0.01 | -0.31 |
| South Africa | -0.15 | 0.06 | -0.26 |
| Eswatini | -0.16 | 0.13 | -0.39 |
| Afghanistan | -0.16 | 0.07 | -0.36 |
| Nauru | -0.16 | 0.03 | -0.67 |
| C么te d'Ivoire | -0.18 | 0.03 | -0.35 |
| Cambodia | -0.19 | 0.04 | -0.36 |
| Egypt | -0.20 | 0.01 | -0.38 |
| Viet Nam | -0.20 | 0.04 | -0.39 |
| Georgia | -0.21 | 0.72 | -0.37 |
| Albania | -0.21 | 0.03 | -0.39 |
| Senegal | -0.21 | 0.00 | -0.38 |
| Zambia | -0.22 | 0.01 | -0.45 |
| Somalia | -0.22 | 0.07 | -0.41 |
| Yemen | -0.22 | 0.03 | -0.41 |
| Kazakhstan | -0.22 | 0.05 | -0.35 |
| Uganda | -0.23 | -0.06 | -0.39 |
| Lao People's Democratic Republic | -0.24 | -0.02 | -0.41 |
| Serbia | -0.24 | 0.18 | -0.40 |
| Madagascar | -0.24 | -0.03 | -0.43 |
| Malawi | -0.25 | -0.06 | -0.40 |
| Namibia | -0.27 | -0.02 | -0.44 |
| Kenya | -0.27 | -0.12 | -0.44 |
| Bulgaria | -0.27 | -0.05 | -0.45 |
| Angola | -0.27 | -0.03 | -0.43 |
| Nicaragua | -0.28 | -0.15 | -0.43 |
| Venezuela (Bolivarian Republic of) | -0.28 | -0.07 | -0.45 |
| Samoa | -0.28 | -0.03 | -0.68 |
| Haiti | -0.28 | -0.08 | -0.45 |
| Lithuania | -0.28 | 1673572.41 | -4378675563.89 |
| Armenia | -0.28 | -0.07 | -0.40 |
| Morocco | -0.29 | -0.16 | -0.45 |
| Mongolia | -0.30 | -0.09 | -0.48 |
| Iraq | -0.30 | -0.13 | -0.45 |
| India | -0.30 | -0.18 | -0.41 |
| South Sudan | -0.31 | -0.03 | -0.49 |
| Nigeria | -0.31 | -0.05 | -0.50 |
| United Republic of Tanzania | -0.31 | -0.14 | -0.47 |
| Botswana | -0.33 | -0.10 | -0.53 |
| Sudan | -0.34 | -0.17 | -0.47 |
| Paraguay | -0.34 | -0.14 | -0.50 |
| Bolivia (Plurinational State of) | -0.34 | -0.14 | -0.49 |
| Bangladesh | -0.34 | -0.17 | -0.50 |
| Bhutan | -0.35 | -0.11 | -0.52 |
| Mexico | -0.35 | -0.25 | -0.45 |
| Djibouti | -0.35 | -0.13 | -0.54 |
| Syrian Arab Republic | -0.35 | -0.12 | -0.53 |
| Marshall Islands | -0.35 | -0.15 | -0.65 |
| Latvia | -0.35 | 1500634662.64 | -23769565189.43 |
| Bosnia and Herzegovina | -0.36 | -0.21 | -0.49 |
| Tonga | -0.36 | -0.17 | -0.90 |
| Romania | -0.37 | -0.19 | -0.47 |
| American Samoa | -0.37 | -0.25 | -0.95 |
| Micronesia (Federated States of) | -0.37 | -0.15 | -0.64 |
| Jamaica | -0.38 | -0.22 | -0.52 |
| Tunisia | -0.38 | -0.18 | -0.54 |
| Tuvalu | -0.38 | -0.17 | -0.68 |
| Malaysia | -0.38 | -0.21 | -0.54 |
| Ecuador | -0.38 | -0.21 | -0.52 |
| Sri Lanka | -0.39 | 3.93 | -0.54 |
| Estonia | -0.39 | 266382.44 | -5.05 |
| Saudi Arabia | -0.40 | -0.21 | -0.57 |
| Myanmar | -0.41 | -0.24 | -0.53 |
| Tokelau | -0.41 | -0.25 | -0.60 |
| United States Virgin Islands | -0.42 | -0.26 | -0.73 |
| Bahamas | -0.42 | -0.26 | -0.64 |
| Mauritania | -0.42 | -0.24 | -0.54 |
| Eritrea | -0.42 | -0.22 | -0.60 |
| China | -0.42 | -0.30 | -0.53 |
| Uruguay | -0.43 | -0.19 | -0.49 |
| Gabon | -0.43 | -0.27 | -0.61 |
| Cuba | -0.43 | -0.30 | -0.53 |
| Burundi | -0.43 | -0.22 | -0.58 |
| Saint Vincent and the Grenadines | -0.43 | -0.33 | -0.53 |
| Niue | -0.44 | -0.18 | -0.68 |
| Suriname | -0.44 | -0.32 | -0.56 |
| Congo | -0.44 | -0.30 | -0.58 |
| Libya | -0.44 | -0.25 | -0.65 |
| Palau | -0.44 | -0.27 | -0.81 |
| Belize | -0.45 | -0.33 | -0.59 |
| Palestine | -0.45 | -0.29 | -0.58 |
| Andorra | -0.45 | -0.12 | -0.61 |
| Guatemala | -0.45 | -0.31 | -0.56 |
| Guyana | -0.46 | -0.30 | -0.59 |
| Fiji | -0.46 | -0.29 | -0.97 |
| Antigua and Barbuda | -0.46 | -0.36 | -0.60 |
| Dominica | -0.46 | -0.32 | -0.62 |
| Costa Rica | -0.46 | -0.33 | -0.58 |
| San Marino | -0.47 | -0.27 | -0.67 |
| El Salvador | -0.47 | -0.33 | -0.60 |
| Northern Mariana Islands | -0.48 | -0.34 | -1.00 |
| Comoros | -0.48 | -0.16 | -0.65 |
| Qatar | -0.49 | -0.12 | -0.61 |
| Greece | -0.49 | -0.44 | -0.59 |
| Argentina | -0.49 | 1.11 | -0.56 |
| Iran (Islamic Republic of) | -0.50 | -0.45 | -0.57 |
| Panama | -0.51 | -0.38 | -0.63 |
| Croatia | -0.51 | 24.78 | -0.62 |
| Seychelles | -0.52 | -0.37 | -0.74 |
| Thailand | -0.52 | 105.71 | -0.68 |
| Kuwait | -0.52 | -0.41 | -0.64 |
| Algeria | -0.52 | -0.40 | -0.63 |
| Hungary | -0.52 | -0.18 | -0.64 |
| Slovakia | -0.53 | -0.15 | -0.63 |
| Barbados | -0.53 | -0.42 | -0.68 |
| Lebanon | -0.53 | -0.43 | -0.66 |
| Oman | -0.54 | -0.40 | -0.66 |
| Greenland | -0.54 | -0.44 | -0.63 |
| Ethiopia | -0.54 | -0.39 | -0.66 |
| Grenada | -0.55 | -0.47 | -0.63 |
| Jordan | -0.57 | -0.47 | -0.67 |
| Rwanda | -0.57 | -0.46 | -0.67 |
| Chile | -0.57 | 9.37 | -2.81 |
| Peru | -0.58 | -0.43 | -0.69 |
| Cook Islands | -0.58 | -0.43 | -0.82 |
| Guam | -0.58 | -0.42 | -1.00 |
| Monaco | -0.59 | -0.46 | -0.73 |
| Poland | -0.59 | -0.50 | -0.67 |
| United Arab Emirates | -0.61 | -0.46 | -0.81 |
| Saint Lucia | -0.62 | -0.53 | -0.71 |
| Malta | -0.62 | -0.57 | -0.66 |
| Turkey | -0.62 | -0.51 | -0.74 |
| Switzerland | -0.62 | -0.22 | -0.68 |
| Sweden | -0.62 | -0.48 | -0.79 |
| Equatorial Guinea | -0.62 | -0.47 | -0.73 |
| Slovenia | -0.62 | -0.18 | -0.73 |
| Brazil | -0.62 | -0.58 | -0.67 |
| Germany | -0.63 | 0.85 | -0.72 |
| Spain | -0.63 | -0.59 | -0.69 |
| Trinidad and Tobago | -0.64 | -0.52 | -0.86 |
| Brunei Darussalam | -0.64 | -0.55 | -0.79 |
| Austria | -0.64 | -0.53 | -0.74 |
| Saint Kitts and Nevis | -0.64 | -0.57 | -0.74 |
| Belgium | -0.65 | -0.61 | -0.69 |
| Taiwan (Province of China) | -0.65 | -0.56 | -0.75 |
| Cyprus | -0.65 | -0.59 | -0.73 |
| Puerto Rico | -0.65 | -0.54 | -0.82 |
| Czechia | -0.66 | -0.39 | -0.73 |
| Italy | -0.66 | -0.59 | -0.75 |
| United States of America | -0.66 | -0.57 | -0.82 |
| Maldives | -0.66 | -0.60 | -0.72 |
| Mauritius | -0.66 | -0.57 | -0.78 |
| Finland | -0.66 | 0.71 | -3.14 |
| New Zealand | -0.67 | -0.64 | -0.73 |
| France | -0.68 | -0.63 | -0.77 |
| Colombia | -0.69 | -0.61 | -0.77 |
| Canada | -0.70 | -0.58 | -0.91 |
| Bahrain | -0.70 | -0.63 | -0.77 |
| Bermuda | -0.71 | -0.65 | -0.79 |
| Denmark | -0.72 | 219099556601.61 | -153082951102.28 |
| Australia | -0.72 | -0.69 | -0.77 |
| Iceland | -0.72 | -0.66 | -0.83 |
| Netherlands | -0.72 | -0.68 | -0.83 |
| Portugal | -0.73 | -0.70 | -0.77 |
| Norway | -0.74 | -0.70 | -0.81 |
| Japan | -0.74 | -0.66 | -0.89 |
| United Kingdom | -0.75 | -0.70 | -0.83 |
| Luxembourg | -0.75 | -0.70 | -0.84 |
| Singapore | -0.75 | -0.70 | -0.84 |
| Ireland | -0.76 | -0.72 | -0.83 |
| Israel | -0.77 | -0.74 | -0.85 |
| Republic of Korea | -0.84 | -0.81 | -0.89 |

ASR, age-standardized rates; CVD, cardiovascular diseases; DALY, disability-adjusted life-years.
